# New Chromones from a Marine-Derived Fungus, *Arthrinium* sp., and Their Biological Activity

**DOI:** 10.3390/molecules23081982

**Published:** 2018-08-09

**Authors:** Jie Bao, Fei He, Jin-Hai Yu, Huijuan Zhai, Zhi-Qiang Cheng, Cheng-Shi Jiang, Yuying Zhang, Yun Zhang, Xiaoyong Zhang, Guangying Chen, Hua Zhang

**Affiliations:** 1 School of Biological Science and Technology, University of Jinan, 336 West Road of Nan Xinzhuang, Jinan 250022, China; bio_baoj@ujn.edu.cn (J.B.); 18864838287@163.com (F.H.); yujinhai12@sina.com (J.-H.Y.); zhai18363005528@163.com (H.Z.); czq13515312897@163.com (Z.-Q.C.); jiangchengshi-20@163.com (C.-S.J.); yuyingzhang2008@163.com (Y.Z.); 2 Key Laboratory of Tropical Medicinal Plant Chemistry of Ministry of Education, Hainan Normal University, 99 South Road of Longkun Road, Haikou 571158, China; 3 Key Laboratory of Tropical Marine Bio-Resources and Ecology, South China Sea Institute of Oceanology, Chinese Academy of Sciences, 164 West Xingang Road, Guangzhou 510301, China; zhangyun@scsio.ac.cn; 4 College of Marine Sciences, South China Agricultural University, 483 Wushan Road, Guangzhou 510642, China; zhangxiaoyong@scau.edu.cn

**Keywords:** *Arthrinium* sp., chromone, polyketide, antioxidant activity

## Abstract

Five new chromone derivatives, arthones A–E (**1**–**5**), together with eight known biogenetically related cometabolites (**6**–**13**), were isolated from a deep-sea-derived fungus *Arthrinium* sp. UJNMF0008. Their structures were assigned by detailed analyses of spectroscopic data, while the absolute configurations of **1** and **5** were established by electronic circular dichroism (ECD) calculations and that of **2** was determined by modified Mosher ester method. Compounds **3** and **8** exhibited potent antioxidant property with DPPH and ABTS radical scavenging activities, with IC_50_ values ranging from 16.9 to 18.7 μM. Meanwhile, no compounds indicated obvious bioactivity in our antimicrobial and anti-inflammatory assays at 50.0 μM.

## 1. Introduction

The genus *Arthrinium* has wide geographic distribution and host range as plant pathogens, endophytes, saprobes, etc., while Poaceae and Cyperaceae are the major host plant families [1]. Although some *Arthrinium* species have been reported as phytopathogens [2,3,4], or even to cause cutaneous infections in humans [5], many others are known to produce diverse bioactive compounds with a variety of pharmacological applications. For instance, cytotoxic cytochalasins, pyridone alkaloids and polyketides, along with naphthalene glycosides with COX-2 inhibitory activity, were obtained from the sponge-derived fungus *A. arundinis* ZSDS1-F3 [6,7,8,9]; arundifungin with antifungal property was isolated from another *A. arundinis* species [10]; griseofulvin derivatives showing lethality against the brine shrimp *Artemia salina* were reported from a gorgonian-derived *Arthrinium* fungus [11]; antiangiogenic diterpenes were discovered from the marine species *A. sacchari* [12]; anti-parasitic dihydroisocoumarins were found in an endophytic *Arthrinium* sp. from *Apiospora montagnei* [13]; and volatile compounds in endophytic *Arthrinium* sp. MFLUCC16-0042 from *Aquilaria subintegra* were also investigated [14].

During the course of our search for new antibiotics from marine resources, an *Arthrinium* sp. UJNMF0008 from deep-sea sediment gained our interest owing to its strong inhibitory activity against *Staphylococcus aureus*. Subsequent chemical investigation on this species led to the discovery of a series of pyridone alkaloids with antibacterial and cytotoxic activities [15]. In addition to the pyridones, another class of metabolites with strong UV absorption was also revealed by chemical profiling (HPLC & ^1^H-NMR). Further study of the remaining fractions have resulted in the isolation and structural characterization of an array of polyketide compounds, including five new chromone arthones A–E (**1**–**5**) and eight previously reported analogues, AGI-B4 (**6**) [16], 1,3,6-trihydroxy-8-methylxanthone (**7**) [17], 2,3,4,6,8-pentahydroxy-1-methylxanthone (**8**) [18], sydowinin A (**9**) [19], sydowinin B (**10**) [20], conioxanthone A (**11**) [21], engyodontiumone B (**12**) [22], and 8-hydroxy-3-hydroxymethyl-9-oxo-9*H*-xanthene-1-carboxylic acid methyl ester (**13**) [23]. Herein, we describe the details of the isolation, structure elucidation, and biological evaluations of compounds **1**–**13**.

## 2. Results and Discussion

### 2.1. Structure Elucidation

**Figure 1:**
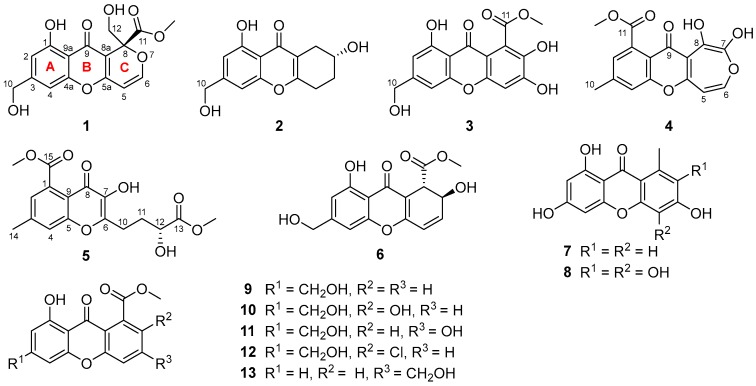
Chemical structures of compounds **1**–**13**.

**Figure 2:**
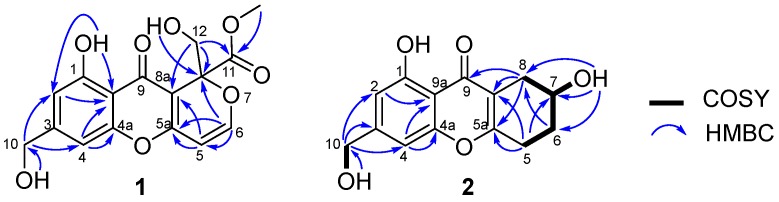
Key 2D-NMR correlations for **1** and **2**.

**Figure 3:**
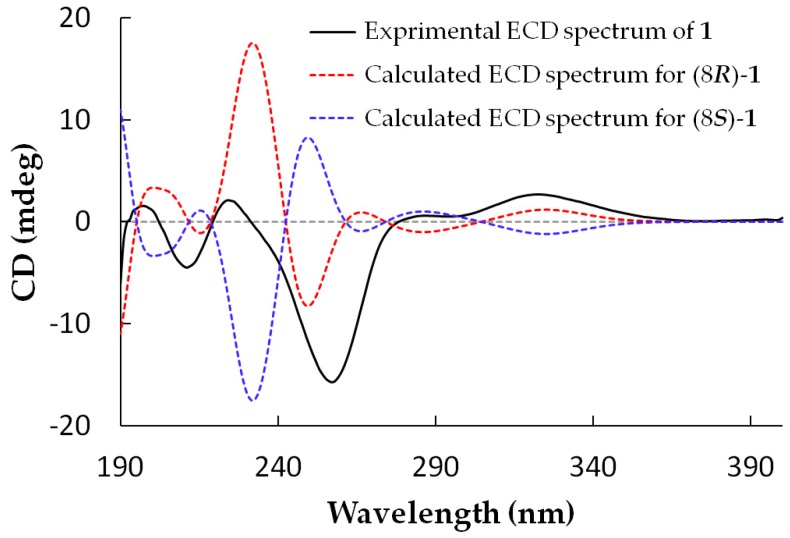
Experimental and theoretical ECD spectra for **1**.

Arthone A (**1**) was isolated as a pale yellow powder and its molecular formula was established as C_16_H_14_O_8_ by HR-ESIMS analysis (*m*/*z* 333.0623 [M − H]^−^) and NMR data. Detailed analysis of the ^1^H- and ^13^C-NMR data (Table 1 and Table 2) showed that **1** possessed the same A and B rings (5-hydroxy-7-(hydroxymethyl)-4*H*-chromen-4-one, Figure 1) moiety as those in **9**–**12** [19,20,21,22], which was further confirmed by the HMBC correlations from 10-O*H* to C-10, H-10 to C-2, C-3 and C-4, H-4 to C-4a, C-9a and C-10, and 1-O*H* to C-1, C-2 and C-9a (Figure 2). Two groups of olefinic signals (δ_H_/δ_C_ 5.72 (*J* = 5.9 Hz)/95.1; 7.33 (*J* = 5.9 Hz)/157.1) including one oxygenated (δ_C_ 157.1, C–6) and one oxyquaternary sp^3^ carbon (δ_C_ 84.3, C-8) revealed similar features (C ring, Figure 1) to the known compound euparvione [24] with the absence of one methyl (δ_H_/δ_C_ 2.06/20.4 in euparvione), along with the appearance of a methyl ester moiety (δ_H_/δ_C_ 3.70/52.8, 167.9) and one hydroxymethyl group (δ_H_/δ_C_ 3.88, 4.12/63.6). Further HMBC correlations from H-12 to C-8, C-8a and C-11, and OC*H*_3_ to C-11 defined the structure of C ring as shown. The planar structure of **1** was thus established with only one chiral center. The absolute configuration of **1** was established as 8*R* by comparison of its experimental and theoretical ECD spectra (Figure 3) [25].

**Figure 4:**
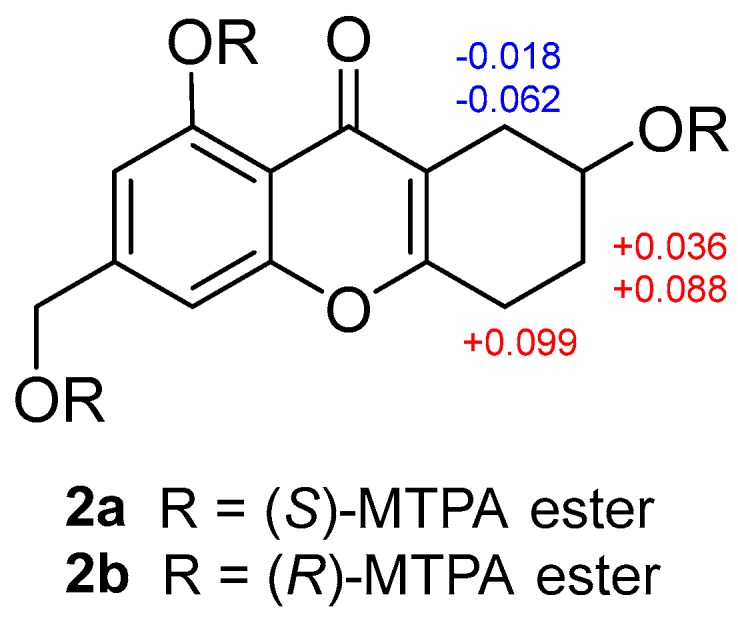
Δδ (δ_S_ − δ_R_) values in ppm for MTPA eaters of **2**.

Arthone B (**2**) was obtained as a pale yellow powder. The molecular formula was deduced as C_14_H_14_O_5_ based on the HR-ESIMS ion at *m*/*z* 263.0916 [M + H]^+^ (calcd. for C_14_H_15_O_5_, 263.0914), indicating eight degrees of unsaturation. 1D-NMR data analysis (Table 1 and Table 2) revealed that **2** also had a 5-hydroxy-7-(hydroxymethyl)-4*H*-chromen-4-one fragment but with larger chemical shifts for C-5a (δ_C_ 165.3) and C-8a (δ_C_ 114.1) compared with those of **1** (δ_C_ 160.6 and 103.8 for C-5a and C-8a, respectively). Correlated spectroscopy (COSY) correlations from H-7 (δ_H_ 3.99) to H_2_-6 (δ_H_ 1.80/1.87) and H_2_-8 (δ_H_ 2.32/2.61), and H-5 (δ_H_ 2.71/2.79) to H-6 (δ_H_ 1.80/1.87), revealed the spin-coupling system from H_2_-5–H_2_-8 (Figure 2). Key HMBC correlations from H-8 to C-5a, C-8a, and C-9 indicated the connection of C-8 to C-8a, while those from H-5 to C-5a and C-8a identified the connection of C-5 to C-5a (Figure 2). The gross structure of **2** was thus characterized, also bearing only one stereocenter. The absolute configuration of **2** was established by modified mosher ester method [26], where analysis of the ^1^H-NMR differences between its (R)- and (S)-MTPA esters (Δδ = δ_S_ − δ_R_) led to the assignment of 7*R*-configuration for **2** (Figure 4).

Arthone C (**3**) was yielded as a yellow powder and displayed an HR-ESIMS peak at *m*/*z* 333.0607 [M + H]^+^ (calcd. for C_16_H_13_O_8_, 333.0605) corresponding to the molecular formula C_16_H_12_O_8,_ with 16 amu more than that of its cometabolite sydowinin B (**10**) [20]. A detailed comparison of their ^1^H- and ^13^C-NMR data (Table 1 and Table 2) revealed that **3** incorporated the same skeleton as that of sydowinin B (**10**) [20] with the downfield-shifted chemical shift of C-6 (δ_C_ 151.2) and upfield-shifted chemical shift of C-5 (δ_C_ 102.5) and C-7 (δ_C_ 141.4), implying the presence of a hydroxyl at C-6, and further HMBC correlations from H-5 to C-6, C-7, and C-8a confirmed this moiety. As mentioned above, the structure of compound **3** was thus elucidated, as shown in Figure 1.

**Figure 5:**
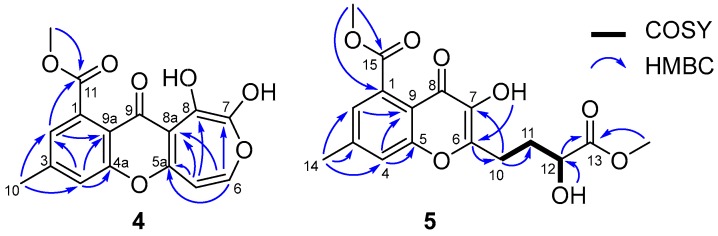
Key 2D-NMR correlations for **4** and **5**.

Arthone D (**4**) was isolated as a yellow powder with the molecular formula of C_16_H_12_O_7_ (11 degrees of unsaturation) determined by (+)-HR-ESIMS analysis at *m*/*z* 317.0664 ([M + H]^+^, calcd. for C_16_H_13_O_7_, 317.0656). The ^1^H- and ^13^C-NMR spectroscopic data for **4** (Table 1 and Table 2) exhibited a similar skeleton to that of isofusidienol A [27]. However, the methoxycarbonly moiety (δ_H_/δ_C_ 3.89/52.7, 168.8) and methyl group (δ_H_/δ_C_ 2.49/21.3) were deduced to locate on the same benzene ring as yicathin A [28] based on the HMBC correlations from OC*H*_3_ to C-11, H-2 to C-4, C-10, and C-11, H-4 to C-2, C-10, C-4a and C-9a, and H-10 to C-2, C-3 and C-4, and the 1D-NMR data (Figure 5). A proton signal at δ_H_ 12.05 in the ^1^H-NMR accounted for a hydroxyl group at C-8 due to H-bonding with C-9 carbonyl. Hence, two olefinic protons (δ_H_ 6.98, d, *J* = 8.9 Hz and 7.34, d, *J* = 8.9 Hz) were speculated to be located on C-5 and C-6, respectively. Finally, the hydroxyl resonanced at δ_H_ 9.45 could only be assigned to C-7. The seven-membered ring moiety was also supported by the HMBC correlations from H-5 to C-5a and C-8a and H-6 to C-7 and C-5a, as well as long range *J*^4^ correlations from H-5 to C-8 and H-6 to C-8a.

Arthone E (**5**) was isolated as a pale yellow powder and had a molecular formula of C_17_H_18_O_8_ as inferred from its (+)-HR-ESIMS data at *m*/*z* 351.1073 [M + H]^+^. Its NMR data (Table 1 and Table 2) revealed the same 7-methyl-4-oxo-4*H*-chromene-5-carboxylate moiety as arthone D (**4**). COSY correlations from H-11 (δ_H_ 1.93/2.08) to H-10 (δ_H_ 2.84) and H-12 (δ_H_ 4.14), and 12-OH (δ_H_ 5.63) to H-12 (δ_H_ 4.14), and HMBC correlations from OC*H*_3_ to C-13, and H-11/H-12 to C-13, revealed the moiety of C-10–C-13–OCH_3_ (Figure 5). Finally, the side-chain fragment was proved to be located at C-6, as supported by the HMBC correlations from H_2_-10 to C-6 and C-7, and a hydroxyl group was suggested at C-7 as indicated by the HMBC correlation from 7-OH to C-6 (Figure 5). The absolute configuration of **5** was established as 12*R* by comparison of its experimental and theoretical ECD spectra (Supplementary material Figure S31).

All the raw spectroscopic data including 1D/2D NMR and HR-ESIMS spectra for the new compounds **1** (Figures S1–S5), **2** (Figures S6–S11), **3** (Figures S12–S16), **4** (Figures S17–S21) and **5** (Figures S22–S27), and the 1D/2D NMR spectra for MTPA esters of **2** (Figures S28–S30) have been provided in the supplementary material.

### 2.2. Biological Activity

In order to evaluate the biological properties of **1**–**13**, their antioxidant activity was assayed by DPPH and ABTS radical scavenging methods with curcumin as positive control (IC_50_ = 24.3 and 9.5 μM, respectively). Only compounds **3** and **8** exhibited significant antioxidant activities, with IC_50_ values of 16.9 and 22.1 μM for DPPH assay, and 18.7 and 18.0 μM for ABTS assay, respectively, while others showed no significant effect at 50.0 μM. Meanwhile, the antimicrobial activity against Gram-positive bacterial strains *Mycobacterium smegmatis* ATCC 607 and *Staphylococcus aureus* ATCC 25923, Gram-negative *Escherichia coli* ATCC 8739, *Pseudomonas aeruginosa* ATCC 9027, and fungus *Candida albicans* ATCC10231, as well as anti-inflammatory activity based on the inhibition effect of NO production in lipopolysaccharide (LPS)-induced mouse macrophages RAW 264.7 cells, were evaluated for **1**–**13**. However, no compounds displayed obvious bioactivity in the two assays up to 50.0 μM.

## 3. Experimental Section

### 3.1. General Experimental Procedures

NMR spectra were recorded on a Bruker Avance DRX600 NMR spectrometer (Bruker BioSpin AG, Fällanden, Switzerland), with residual solvent peaks as references (DMSO-*d*_6_: δ_H_ 2.50, δ_C_ 39.52). ESIMS analyses were carried out on an Agilent 1260-6460 Triple Quad LC-MS instrument (Agilent Technologies Inc., Waldbronn, Germany). HR-ESIMS data were acquired on an Agilent 6545 Q-TOF mass spectrometer (Agilent Technologies Inc., Waldbronn, Germany). UV spectra were obtained on a Shimadzu UV-2600 spectrophotometer (Shimadzu, Kyoto, Japan) with 1 cm pathway cell. Optical rotations were measured on a Rudolph VI polarimeter (Rudolph Research Analytical, Hackettstown, NJ, USA) with a 10 cm length cell. ECD spectra were acquired on a Chirascan circular-dichroism spectrometer (Applied Photophysics Ltd., Surrey, UK). IR spectra were recorded on an FT-IR VERTEX 70 (Bruker BioSpin AG, Fremont, CA, USA). All HPLC analyses and separations were carried out on Agilent 1260 series LC instruments (Agilent Technologies Inc., Waldbronn, Germany) and a YMC-Pack ODS-A column (250 × 10 mm, 5 μm) was used for HPLC separations. Column chromatography (CC) was performed on Silica gel (200–300 mesh, Yantai Jiangyou Silica Gel Development Co., Yantai, China) and Sephadex LH-20 gel (GE Healthcare Bio-Sciences AB, Uppsala, Sweden). All solvents used for CC were of analytical grade (Tianjin Fuyu Fine Chemical Co. Ltd., Tianjin, China) and solvents used for HPLC were of HPLC grade (Oceanpak Alexative Chemical Ltd., Goteborg, Sweden).

### 3.2. Fungal Material

The fungus strain UJNMF0008 was isolated from a marine-sediment sample collected in the South China Sea (17°55′00″ N, 115°55′31″ E; 3858 m depth, Hainan, China). This strain was identified as an *Arthrinium* sp. based on morphological traits and a molecular biological protocol by DNA amplification and comparison of its ITS region sequence with the GenBank database (100% similarity with *Arthrinium* sp. zzz1842 (HQ696050.1)). The BLAST sequenced data were deposited at GenBank (No. MG010382). The strain was deposited at the CGMCC center, Institute of Microbiology, Chinese Academy of Sciences (Beijing, China).

### 3.3. Fermentation and Extraction

*Arthrinium* sp. UJNMF0008 from a PDA culture plate was inoculated in 500 mL Erlenmeyer flasks containing 150 mL soluble-starch medium (1% glucose, 0.1% soluble starch, 1% MgSO_4_, 0.1% KH_2_PO_4_, 0.1% peptone, and 3% sea salt) at 28 °C on a rotary shaker at 180 rpm for 3 days as seed cultures. Then, each of the seed cultures (20 mL) was transferred into autoclaved 1 L Erlenmeyer flasks with solid-rice medium (each flasks contained 80 g commercially available rice, 0.4 g yeast extract, 0.4 g glucose, and 120 mL water with 3% sea salt). After that, the strain was incubated statically for 30 days at 28 °C.

After fermentation, the total 4.8 kg rice culture was crushed and extracted with 15.0 L 95% EtOH three times. The EtOH extract was evaporated under reduced pressure to afford an aqueous solution and then extracted with 2.0 L ethyl acetate three times to give 80 g crude gum.

### 3.4. Isolation and Purification

The ethyl acetate extract (80 g) was fractionated by a silica gel column eluting with step gradient CH_2_Cl_2_-MeOH (*v*/*v* 100:0, 98:2, 95:5, 90:10, 80:20, 70:30, 50:50 and 0:100) to give 10 fractions (Fr.1–Fr.10) based on TLC and HPLC analysis. Fr.6 (30.2 g) was applied to CC over D101-macroporous absorption resin eluted with EtOH-H_2_O (30%, 50%, 80% and 100%) to afford four subfractions (Fr.6-1–Fr.6-4). Fr.6-3 (11.6 g) was fractionated by the silica gel column with step gradient CH_2_Cl_2_-(CH_3_)_2_CO (*v*/*v* 100:0–0:100) and divided into nine subfractions (Fr.6-3-1–Fr.6-3-9) and a portion of (32.5 mg) Fr.6-3-3 was further purified by HPLC eluting with MeOH-H_2_O (*v*/*v* 55:45, 3.0 mL min^−1^) to give **13** (*t*_R_ = 29.3 min, 20.6 mg). Fr.6-3-2 (0.67 g), Fr.6-3-4 (510 mg), Fr.6-3-5 (1.04 g), Fr.6-3-6 (3.9 g), and Fr.6-3-7 (2.2 g) were separated by MPLC with an ODS column eluting with gradient MeOH-H_2_O (*v*/*v* 20:80–100:0) to obtain five, five, six, six and five subfractions, respectively. Fr.6-3-2-4 (37.0 mg) was further purified by HPLC eluting with MeOH-H_2_O (*v*/*v* 55:45, 3.0 mL min^−1^) to give **9** (*t*_R_ = 22.2 min, 9.8 mg). Fr.6-3-4-3 (37.0 mg) was further purified by HPLC eluting with MeOH-H_2_O-CH_3_CO_2_H (*v*/*v*/*v* 70:30:10^−4^, 3.0 mL min^−1^) to afford **4** (*t*_R_ = 14.4 min, 1.8 mg), while Fr.6-3-4-5 (50.2 mg) was further purified by HPLC eluting with MeOH-H_2_O (*v*/*v* 84:16, 3.0 mL min^−1^) to give **7** (*t*_R_ = 21.9 min, 21.0 mg). Fr.6-3-5-2 (25.0 mg) was further purified by HPLC eluting with MeOH-H_2_O (*v*/*v* 48:52, 3.0 mL min^−1^) to give **1** (*t*_R_ = 13.9, 4.5 mg), while Fr.6-3-5-5 (35.2 mg) was further purified by HPLC eluting with MeOH-H_2_O (*v*/*v* 60:40, 3.0 mL min^−1^) to give **10** (*t*_R_ = 7.9 min, 4.0 mg) and **11** (*t*_R_ = 16.4 min, 11.7 mg), and Fr.6-3-5-6 (25.1 mg) was further purified by HPLC eluting with MeOH-H_2_O (*v*/*v* 65:35, 3.0 mL min^−1^) to give **12** (*t*_R_ = 20.7 min, 2.4 mg). Fr.6-3-6-2 (55.2 mg) was further purified by HPLC eluting with MeOH-H_2_O (*v*/*v* 34:66, 3.0 mL min^−1^) to give **6** (*t*_R_ = 36.9 min, 14.3 mg). Fr.6-3-7-2 (80.1 mg) was further purified by HPLC eluting with MeOH-H_2_O (*v*/*v* 42:58, 3.0 mL min^−1^) to give **2** (*t*_R_ = 11.1 min, 2.7 mg). Fr.7 (15.2 g) was chromatographed on a silica gel column with step gradient CH_2_Cl_2_-(CH_3_)_2_CO (*v*/*v* 100:0–0:100) and divided into three subfractions (Fr.7-1–Fr.7-3). Fr.7-1 (3.5 g) was divided into three subfractions (Fr.7-1-1–Fr.7-1-3) by Sephadex LH-20 CC eluting with MeOH-CH_2_Cl_2_ (*v*/*v* 1:1), and Fr.7-1-2 (1.2 g) was fractionated by MPLC with an ODS column eluting with step gradient MeOH-H_2_O (*v*/*v* 20:80 to 0:100) and further purified by HPLC eluting with MeOH-H_2_O (*v*/*v* 34:66, 3.0 mL min^−1^) to provide **5** (*t*_R_ = 46.9 min, 2.6 mg). Fr.9 (7.6 g) was subject to silica gel column with step gradient CH_2_Cl_2_-(CH_3_)_2_CO (*v*/*v* 100:0–0:100) and divided into five subfractions (Fr.9-1–Fr.9-5). Fr.9-2 (1.6 g) was separated by Sephadex LH-20 CC eluting with MeOH-CH_2_Cl_2_ (*v*/*v* 1:1) to obtain four subfractions (Fr.9-2-1–Fr.9-2-4), and Fr.9-2-2 (42.6 mg) was further purified by HPLC eluting with MeOH-H_2_O-CH_3_CO_2_H (*v*/*v*/*v* 54:46:10^−4^, 3.0 mL min^−1^) to give **3** (*t*_R_ = 17.1 min, 9.3 mg), while Fr.9-2-3 (17.0 mg) was further purified by HPLC eluting with MeOH-H_2_O (*v*/*v* 54:46, 3.0 mL min^−1^) to give **8** (*t*_R_ = 26.4 min, 3.7 mg).

#### 3.4.1. Arthone A (**1**)

Pale yellow powder; ${\left[\alpha \right]}_{D}^{23}$ −6.3 (*c* 0.56, MeOH); ECD (0.20 mg mL^−1^, MeOH) *λ* (Δε) 322 (2.76), 291 (0.56), 258 (−15.78), 224 (2.50), 212 (−5.55), 204 (1.71) nm; UV (MeOH) *λ*_max_ (log ε) 238 (4.35), 259 (4.23), 331 (3.64) nm; IR (KBr) *ν*_max_ 3404, 2957, 1742, 1658, 1594, 1493, 1451, 1299, 1195, 1051, 1022, 821 cm^−1^; ^1^H- and ^13^C-NMR data, Table 1 and Table 2; (−)-ESIMS *m*/*z* 332.9 [M − H]^−^; (−)-HR-ESIMS *m*/*z* 333.0623 [M − H]^−^ (calcd. for C_16_H_13_O_8_, 333.0616).

#### 3.4.2. Arthone B (**2**)

Pale yellow powder; ${\left[\alpha \right]}_{D}^{23}$ 1.9 (*c* 0.16, MeOH); ECD (0.20 mg mL^−1^, MeOH) *λ* (Δε) 328 (0.42), 286 (0.01), 276 (0.07), 258 (−0.29), 245 (−0.01), 206 (−2.21) nm; UV (MeOH) *λ*_max_ (log ε) 239 (5.18), 328 (4.35) nm; IR (KBr) *ν*_max_ 3431, 2935, 1659, 1625, 1597, 1499, 1459, 1292, 1117, 1041 cm^−1^; ^1^H- and ^13^C-NMR data, Table 1 and Table 2; (−)-ESIMS *m*/*z* [M − H]^−^ 260.9; (+)-HR-ESIMS *m*/*z* 263.0916 [M + H]^+^ (calcd. for C_14_H_15_O_5_, 263.0914).

#### 3.4.3. Arthone C (**3**)

Yellow powder; UV (MeOH) *λ*_max_ (log ε) 233 (4.20), 246 (4.14), 254 (4.13), 296 (3.69), 377 (4.01) nm; IR (KBr) *ν*_max_ 3307, 1702, 1651, 1607, 1586, 1499, 1436, 1381, 1366, 1286, 1250, 1011, 830 cm^−1^; ^1^H- and ^13^C-NMR data, Table 1 and Table 2; (−)-ESIMS *m*/*z* 330.9 [M − H]^−^; (+)-HR-ESIMS *m*/*z* 333.0607 [M + H]^+^ (calcd. for C_16_H_13_O_8_, 333.0605).

#### 3.4.4. Arthone D (**4**)

Yellow powder; UV (MeOH) *λ*_max_ (log ε) 234 (4.27), 267 (4.26), 391 (3.33) nm; IR (KBr) *ν*_max_ 3427, 1725, 1613, 1501, 1459, 1435, 1297, 1235, 1041 cm^−1^; ^1^H- and ^13^C-NMR data, Table 1 and Table 2; (−)-ESIMS *m*/*z* 314.9 [M − H]^−^; (+)-HR-ESIMS *m*/*z* 317.0664 [M + H]^+^ (calcd. for C_16_H_13_O_7_, 317.0656).

#### 3.4.5. Arthone E (**5**)

Pale yellow powder; ${\left[\alpha \right]}_{D}^{23}$ −4.8 (*c* 0.73, MeOH); UV (MeOH) *λ*_max_ (log ε) 209 (4.30), 314 (3.64) nm; IR (KBr) *ν*_max_ 3413, 2957, 1736, 1611, 1438, 1309, 1227, 1176, 1040, 1037, 861 cm^−1^; ^1^H- and ^13^C-NMR data, Table 1 and Table 2; (+)-ESIMS *m*/*z* 351.0 [M + H]^+^; (+)-HR-ESIMS *m*/*z* 351.1073 [M + H]^+^ (calcd. for C_17_H_19_O_8_, 351.1074).

### 3.5. Antioxidant Assay

The antioxidant activities for compounds **1**–**13** were determined by DPPH and ABTS methods. DPPH radical scavenging method was conducted as described formerly [29], while ABTS radical scavenging assay was performed according to the method developed by Re et al. [30] with some modifications as below. Briefly, an ABTS radical solution was prepared by mixing equal volumes of aqueous solutions of 7 mM ABTS and 4.9 mM potassium persulfate for 16 h in the dark at room temperature. Then the ABTS radical solution was diluted with EtOH to an absorbance of 0.70 ± 0.02 at 734 nm. 10 μL of the tested compounds in ethanol (final concentrations as 3.13 μM, 6.25 μM, 12.5 μM, 25.0 μM, 50.0 μM, and 100 μM) was mixed with 190 μL of the prepared diluted ABTS radical solution at room temperature, and the absorbance at 734 nm was measured after 6 min in the dark. IC_50_ values were defined as the concentrations of tested compounds resulting in 50% loss of the ABTS radical. All determinations were carried out in triplicate, and curcumin was applied as positive control.

### 3.6. Antimicrobial Assays

The antimicrobial activity of compounds **1**–**13** was assayed against the Gram-positive bacterial strains *Mycobacterium smegmatis* ATCC 607 and *Staphylococcus aureus* ATCC 25923, Gram-negative *Escherichia coli* ATCC 8739, *Pseudomonas aeruginosa* ATCC 9027, and fungus *Candida albicans* ATCC10231 by the two-fold serial dilution method in 96-well microplates as described previously [15]. Penicillin was used as positive control in the current assay.

### 3.7. Anti-Inflammatory Assay

Determination of nitric oxide production. Briefly, RAW 264.7 cells were plated into 96-well plates and pretreated with a series of concentrations of compounds (3.13, 6.25, 12.5, 25.0, 50.0, and 100 μM) for 1 h before treatment with 1 μg mL^−1^ LPS. After 24 h incubation, detection of accumulated nitric oxide in the cell supernatants was assayed by Griess reagent kit (Beyotime Institute of Biotechnology, Jiangsu, China) according to the manufacturer’s instructions. Equal volumes of culture supernatant and Griess reagent were mixed, and the absorbance at 540 nm was measured using a Microplate Reader (Tecan, Grödig, Austria).

Cell viability assay. RAW 264.7 cells were seeded into 96-well plates at 1 × 10^4^ cells well^−1^ and allowed to attach for 24 h. The medium was replaced with a 100 μL medium containing the indicated concentrations of compounds and further incubated for 24 h. 10 μL of MTT (5 mg mL^−1^ in PBS) was added into each well and the plates were incubated for 4 h at 37 °C. Supernatants were aspirated and formed formazan was dissolved in 100 μL of dimethyl sulfoxide (DMSO). The optical density (OD) was measured at an absorbance wavelength of 490 nm using a Microplate Reader (Tecan, Switzerland).

### 3.8. ECD Calculations

Conformational analysis within an energy window of 3.0 kcal mol^−1^ was performed using the OPLS3 molecular mechanics force field via the MacroModel [31] panel of Maestro 10.2. The conformers were then further optimized with the software package Gaussian 09 [32] at the CAM-B3LYP/6-311G(d,*p*) level for **1** and B3LYP/6-311G(d,*p*) level for **5**, respectively, and the harmonic vibrational frequencies were also calculated to confirm their stability. Then, the 30 lowest electronic transitions for the obtained conformers in vacuum were calculated using time-dependent density functional theory (TD-DFT) method at the CAM-B3LYP/6-311G(d,*p*) level for **1** and B3LYP/6-311G(d,*p*) level for **5**, respectively. ECD spectra of the conformers were simulated using a Gaussian function with a half-bandwidth of 0.25 eV for **1** and 0.35 eV for **5**. The overall theoretical ECD spectra were obtained according to the Boltzmann weighting of each conformer.

## Figures and Tables

**Table 1:** ^1^H-NMR (600 MHz) data for **1**–**5** (DMSO-*d*_6_).

Position	1	2	3	4	Positon	5
2	6.74, brs	6.70, s	6.70, brs	7.27, d (0.9)	2	7.23, brs
4	6.96, brs	6.91, s	6.94 ^a^, brs	7.56, brs	4	7.54, brs
5	5.72, d (5.9)	2.79, dt (18.4, 6.4) 2.71, dt (18.4, 6.4)	6.94 ^a^, s	6.98, d (8.9)	10	2.84, t (7.9)
6	7.33, d (5.9)	1.87, m 1.80, m		7.34, d (8.9)	11	2.08, m 1.93, m
7		3.99, m			12	4.14, m
8		2.61, dd (16.4, 4.2) 2.32, dd (16.4, 5.8)			14	2.44, brs
10	4.56, brd (5.6)	4.54, d (5.8)	4.56, s	2.49, s	13-OCH_3_	3.62, s
12	4.12, dd (12.5, 5.8) 3.88, dd (12.5, 7.2)				15-OCH_3_	3.83, s
OCH_3_	3.70, s		3.83, s	3.89, s	7-OH	9.00, brs
1-OH	12.37, s	12.67, s	12.55, s		12-OH	5.63, d (4.3)
7-OH		4.92, d (3.7)		9.45, s		
8-OH				12.05, s		
10-OH	5.50, t (5.6)	5.47, t (5.8)				
12-OH	5.29, dd (7.2, 5.8)					

^a^ Interchangeable assignments.

**Table 2:** ^13^C-NMR (150 MHz) data for **1**–**5** (DMSO-*d*_6_).

Position	1	2	3	4	Position	5
1	159.4, C	159.5, C	160.5, C	132.8, C	1	132.7, C
2	108.5, CH	107.6, CH	107.1, CH	124.0, CH	2	124.6, CH
3	152.0, C	151.8, C	152.9, C	147.7 ^b^, C	3	144.4, C
4	104.1, CH	103.8, CH	103.8, CH	119.1, CH	4	119.6, CH
4a	154.7, C	155.6, C	155.3, C	156.0, C	5	155.0, C
5	95.1, CH	25.0, CH_2_	102.5, CH	106.3, CH	6	152.7, C
5a	160.6,C	165.3, C	155.2, C	147.4 ^b^, C	7	138.8, C
6	157.1, CH	28.80 ^a^, CH_2_	151.2, C	124.6, CH	8	171.1, C
7		62.9, CH	141.4, C	147.9 ^b^, C	9	117.0, C
8	84.3, C	28.78 ^a^, CH_2_	117.6, C	140.5, C	10	24.8, CH_2_
8a	103.8, C	114.1, C	108.6, C	108.7, C	11	31.2, CH_2_
9	178.2, C	181.9, C	179.1, C	180.9, C	12	69.7, CH
9a	108.8, C	107.9, C	106.3, C	113.7, C	13	174.6, C
10	62.2, CH_2_	62.3, CH_2_	62.4, CH_2_	21.3, CH_3_	14	21.4, CH_3_
11	167.9, C		166.8, C	168.8, C	15	169.5, C
12	63.6, CH_2_				13-OCH_3_	52.0, CH_3_
OCH_3_	52.8, CH_3_		52.2, CH_3_	52.7, CH_3_	15-OCH_3_	52.8, CH_3_

^a^^,b^ Interchangeable assignments.

## Supplementary Materials

The following are available online. 1D/2D-NMR and HR-ESIMS spectra of compounds **1**–**5**, the 1D/2D NMR spectra for MTPA esters of **2**, along with experimental and theoretical ECD spectra for **5**.
