# Supplementary material for: New Chromones from a Marine-Derived Fungus, Arthrinium sp., and Their Biological Activity
Source: Molecules. 2018 Aug 9;23(8):1982. doi: 10.3390/molecules23081982 (PMC6222336; doi:10.3390/molecules23081982)
Supplement: Supplementary file 1 [file molecules-23-01982-s001.pdf]

Supplementary Information for

New Chromones from a Marine-derived Fungus, *Arthrinium* sp.

Jie Bao [1,2], Fei He [1], Jin-Hai Yu [1], Huijuan Zhai [1], Zhi-Qiang Cheng [1], Cheng-Shi Jiang [1], Yuying Zhang [1], Yun Zhang [3], Xiaoyong Zhang [4], Guangying Chen [2,*] and Hua Zhang [1,*]

[1] *School of Biological Science and Technology, University of Jinan, 336 West Road of Nan Xinzhuang, Jinan 250022, China*
[2] *Key Laboratory of Tropical Medicinal Plant Chemistry of Ministry of Education, Hainan Normal University, 99 South Road of Longkun Road, Haikou 571158, China*
[3] *Key Laboratory of Tropical Marine Bio-Resources and Ecology, South China Sea Institute of Oceanology, Chinese Academy of Sciences, 164 West Xingang Road, Guangzhou 510301, China; zhangyun@scsio.ac.cn*
[4] *College of Marine Sciences, South China Agricultural University, 483 Wushan Road, Guangzhou 510642, China*
* *Correspondence: bio_zhangh@ujn.edu.cn; Tel.: +86-531-8973-6199*
chgying123@163.com; Tel.: +86-898-65889422

List of supplementary information

**Figure S1.** The ^{1}H-NMR spectrum of arthone A (**1**) in DMSO-d_6	**P3**
**Figure S2.** The ^{13}C-NMR spectrum of arthone A (**1**) in DMSO-d_6	**P3**
**Figure S3.** The HSQC spectrum of arthone A (**1**) in DMSO-d_6	**P4**
**Figure S4.** The HMBC spectrum of arthone A (**1**) in DMSO-d_6	**P4**
**Figure S5.** The (−)-HR-ESIMS spectrum of arthone A (**1**)	**P5**
**Figure S6.** The ^{1}H-NMR spectrum of arthone B (**2**) in DMSO-d_6	**P5**
**Figure S7.** The ^{13}C-NMR spectrum of arthone B (**2**) in DMSO-d_6	**P6**
**Figure S8.** The HSQC spectrum of arthone B (**2**) in DMSO-d_6	**P6**
**Figure S9.** The ^{1}H-^{1}H COSY spectrum of arthone B (**2**) in DMSO-d_6	**P7**
**Figure S10.** The HMBC spectrum of arthone B (**2**) in DMSO-d_6	**P7**
**Figure S11.** The (+)-HR-ESIMS spectrum of arthone B (**2**)	**P8**
**Figure S12.** The ^{1}H-NMR spectrum of arthone C (**3**) in DMSO-d_6	**P8**
**Figure S13.** The ^{13}C-NMR spectrum of arthone C (**3**) in DMSO-d_6	**P9**
**Figure S14.** The HSQC spectrum of arthone C (**3**) in DMSO-d_6	**P9**
**Figure S15.** The HMBC spectrum of arthone C (**3**) in DMSO-d_6	**P10**
**Figure S16.** The (+)-HR-ESIMS spectrum of arthone C (**3**)	**P10**
**Figure S17.** The ^{1}H-NMR spectrum of arthone D (**4**) in DMSO-d_6	**P11**

**Figure S18.** The ^{13}C-NMR spectrum of arthone D (**4**) in DMSO-d_6	**P11**
**Figure S19.** The HSQC spectrum of arthone D (**4**) in DMSO-d_6	**P12**
**Figure S20.** The HMBC spectrum of arthone D (**4**) in DMSO-d_6	**P12**
**Figure S21.** The (+)-HR-ESIMS spectrum of arthone D (**4**)	**P13**
**Figure S22.** The ^{1}H-NMR spectrum of arthone E (**5**) in DMSO-d_6	**P13**
**Figure S23.** The ^{13}C-NMR spectrum of arthone E (**5**) in DMSO-d_6	**P14**
**Figure S24.** The HSQC spectrum of arthone E (**5**) in DMSO-d_6	**P14**
**Figure S25.** The ^{1}H-^{1}H COSY spectrum of arthone E (**5**) in DMSO-d_6	**P15**
**Figure S26.** The HMBC spectrum of arthone E (**5**) in DMSO-d_6	**P15**
**Figure S27.** The (+)-HR-ESIMS spectrum of arthone E (**5**)	**P16**
**Figure S28.** The ^{1}H-NMR spectrum of (*R*)-MTPA ester of arthone B (**2**) in $CDCl_3$	**P16**
**Figure S29.** The ^{1}H-^{1}H COSY spectrum of (*R*)-MTPA ester of arthone B (**2**) in $CDCl_3$	**P17**
**Figure S30.** The ^{1}H-NMR spectrum of (*S*)-MTPA ester of arthone B (**2**) in $CDCl_3$	**P17**
**Figure S31.** Experimental and theoretical ECD spectra for **5**	**P18**

Figure S1. The ^{1}H-NMR spectrum of arthone A (**1**) in DMSO-d_6

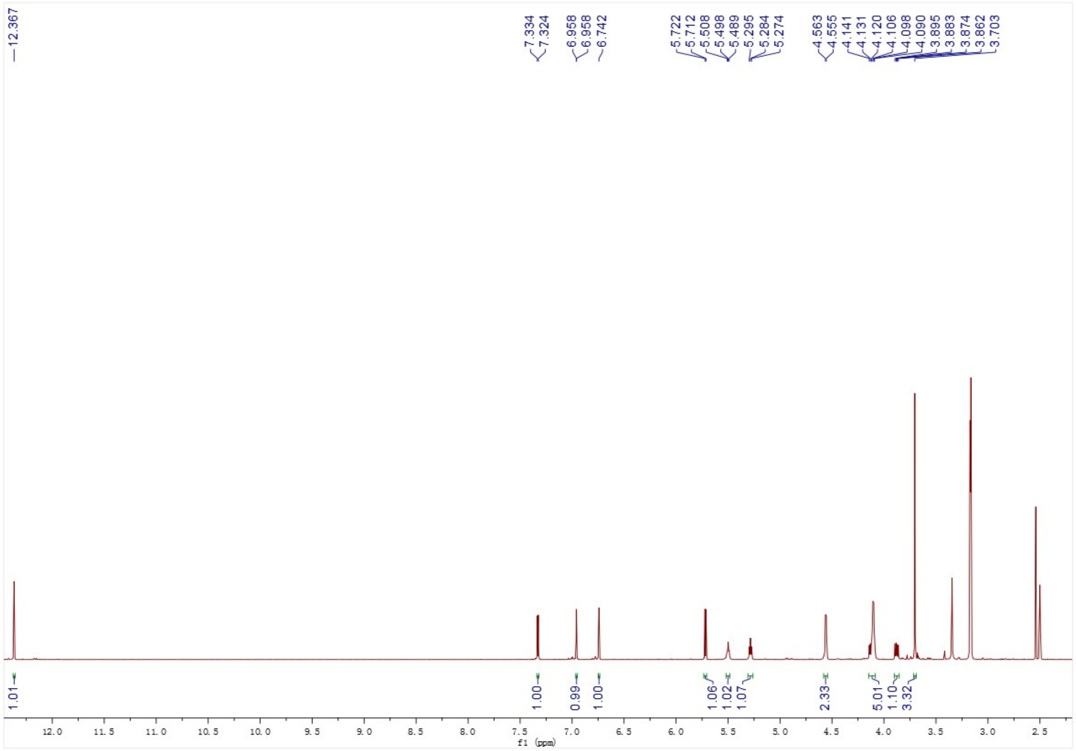

Figure S2. The ^{13}C-NMR spectrum of arthone A (**1**) in DMSO-d_6

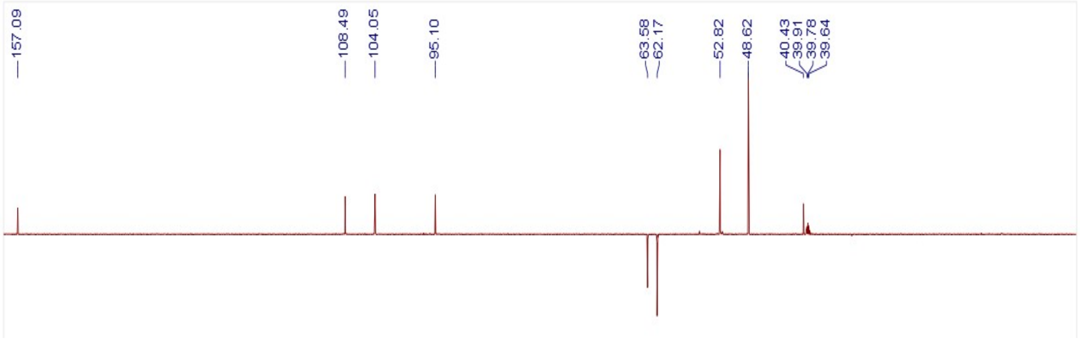

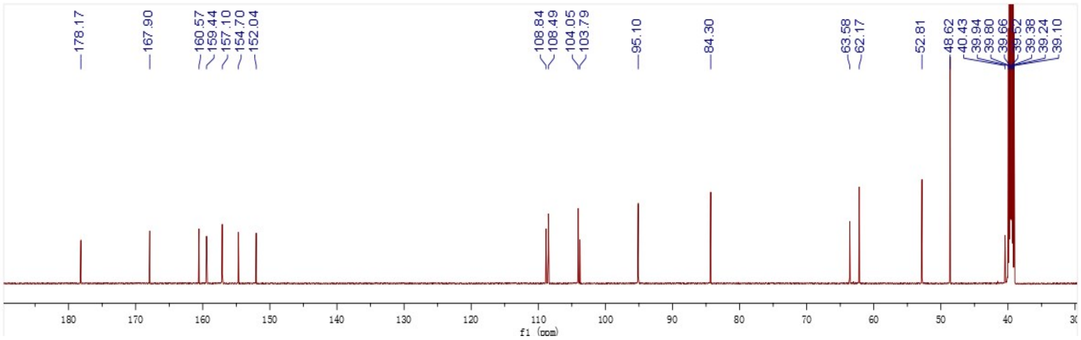

Figure S3. The HSQC spectrum of arthone A (**1**) in DMSO-d_6

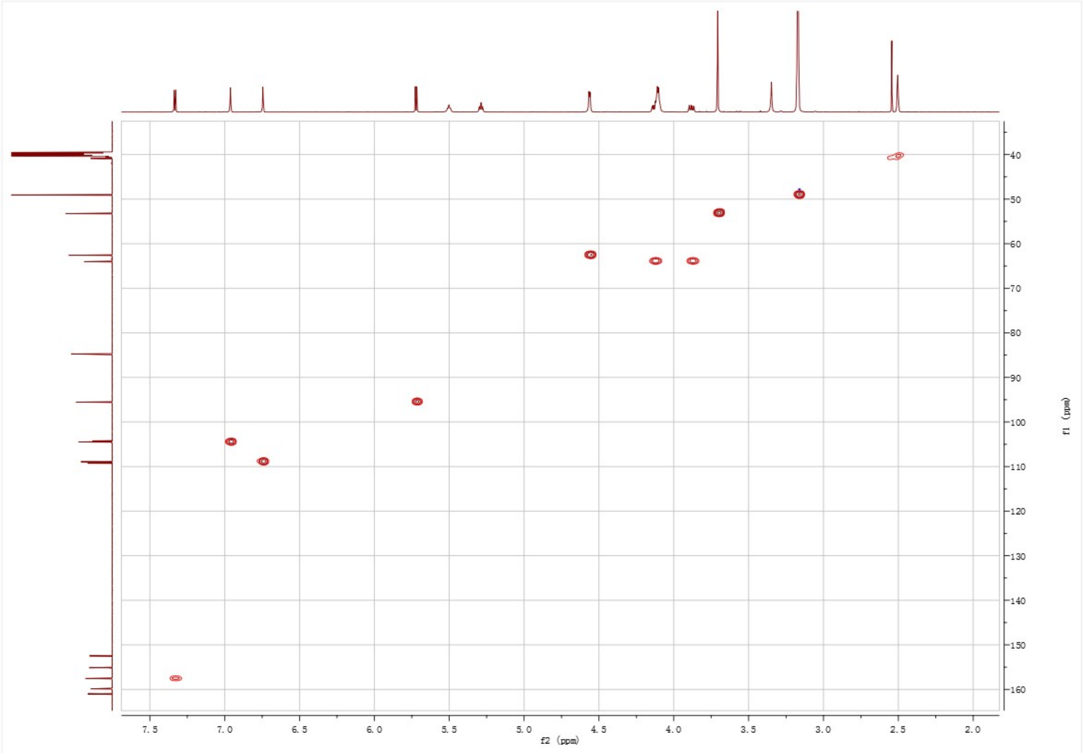

Figure S4. The HMBC spectrum of arthone A (**1**) in DMSO-d_6

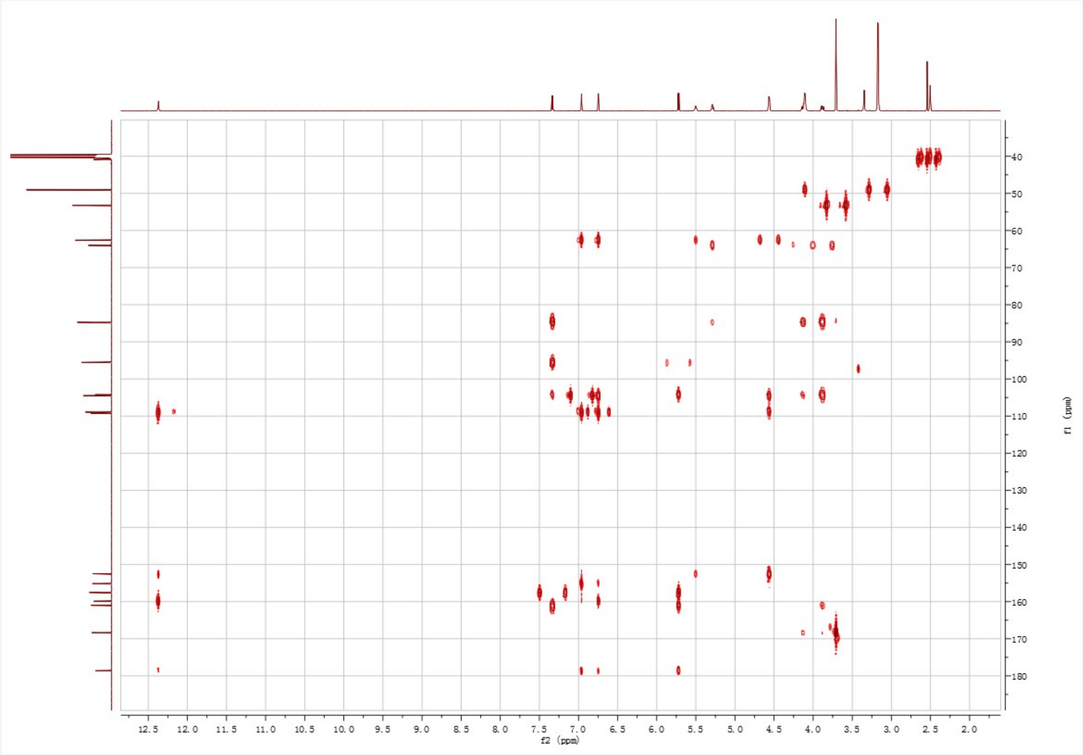

Figure S5. The (−)-HR-ESIMS spectrum of arthone A (**1**)

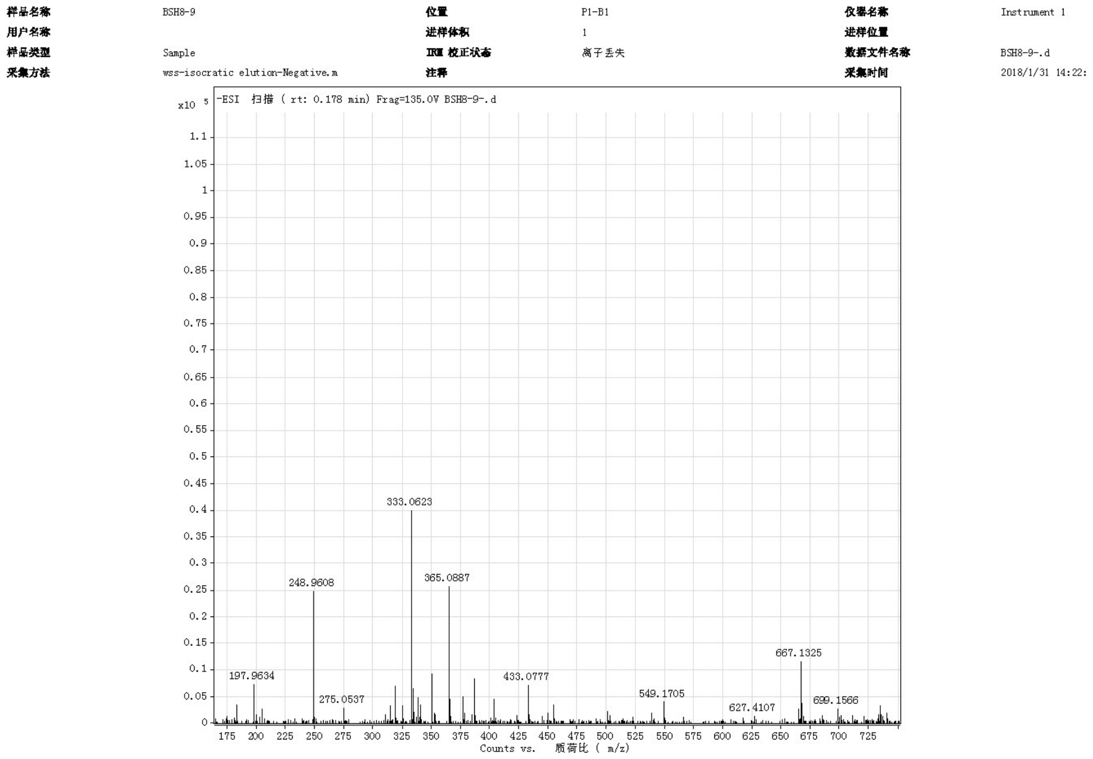

Figure S6. The ^{1}H-NMR spectrum of arthone B (**2**) in DMSO-d_6

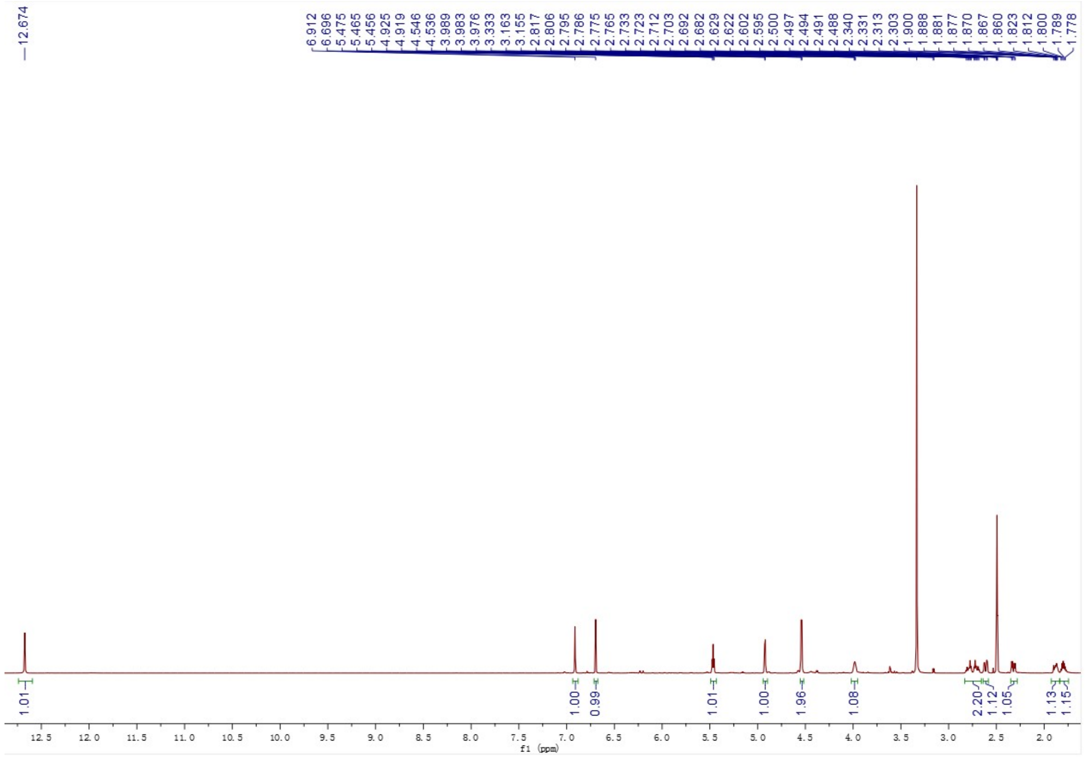

Figure S7. The ^{13}C-NMR spectrum of arthone B (**2**) in DMSO-d_6

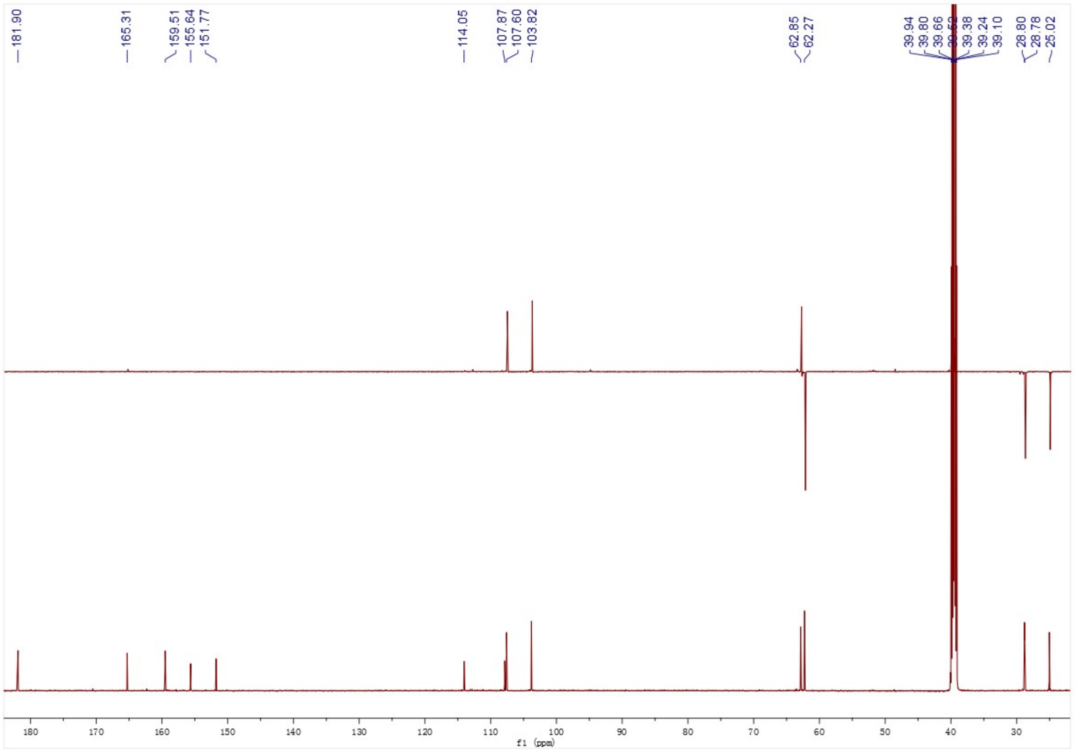

Figure S8. The HSQC spectrum of arthone B (**2**) in DMSO-d_6

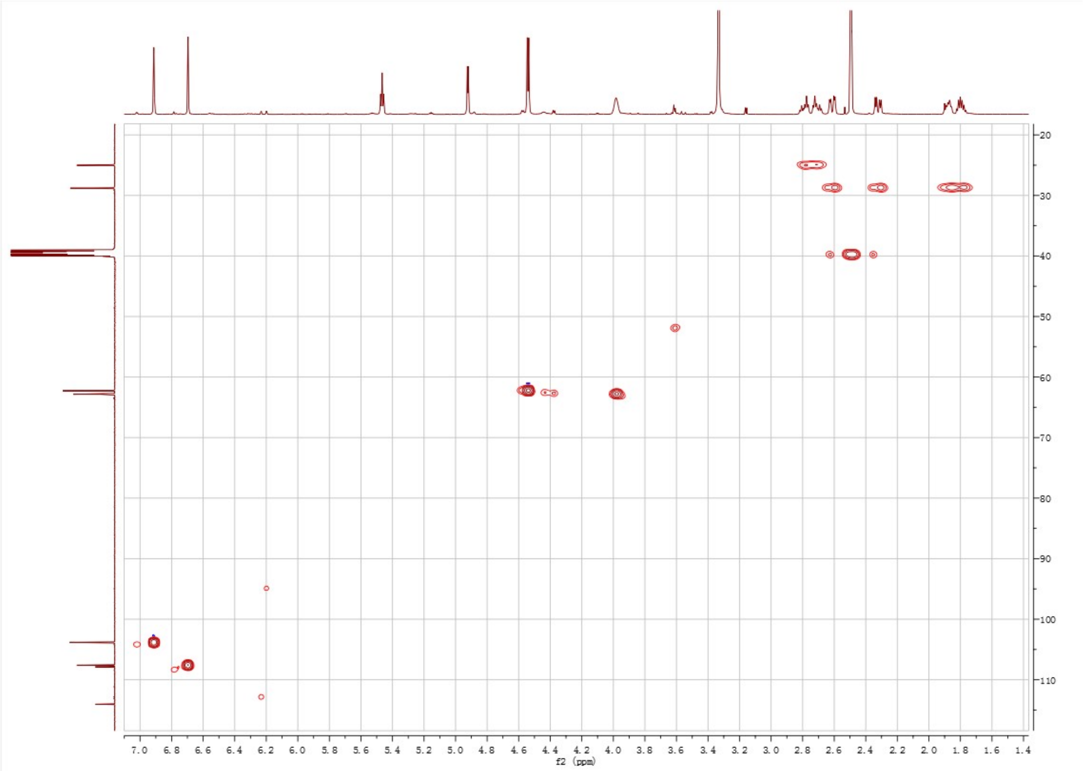

Figure S9. The ^{1}H-^{1}H COSY spectrum of arthone B (**2**) in DMSO-d_6

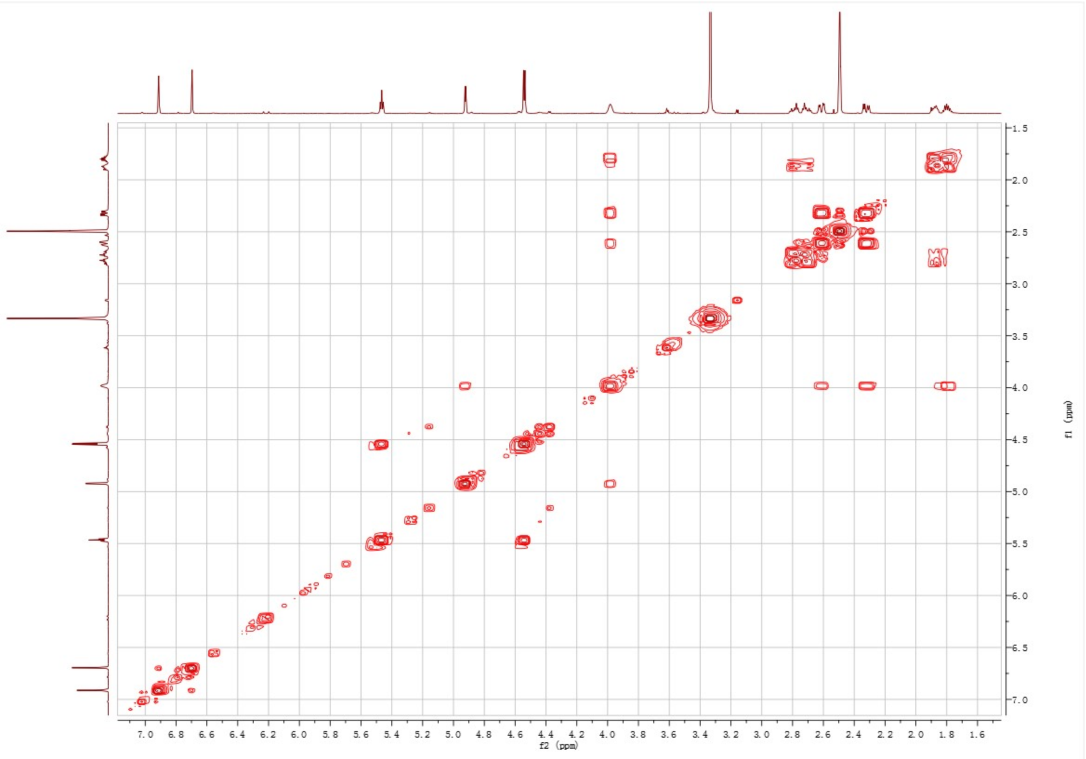

Figure S10. The HMBC spectrum of arthone B (**2**) in DMSO-d_6

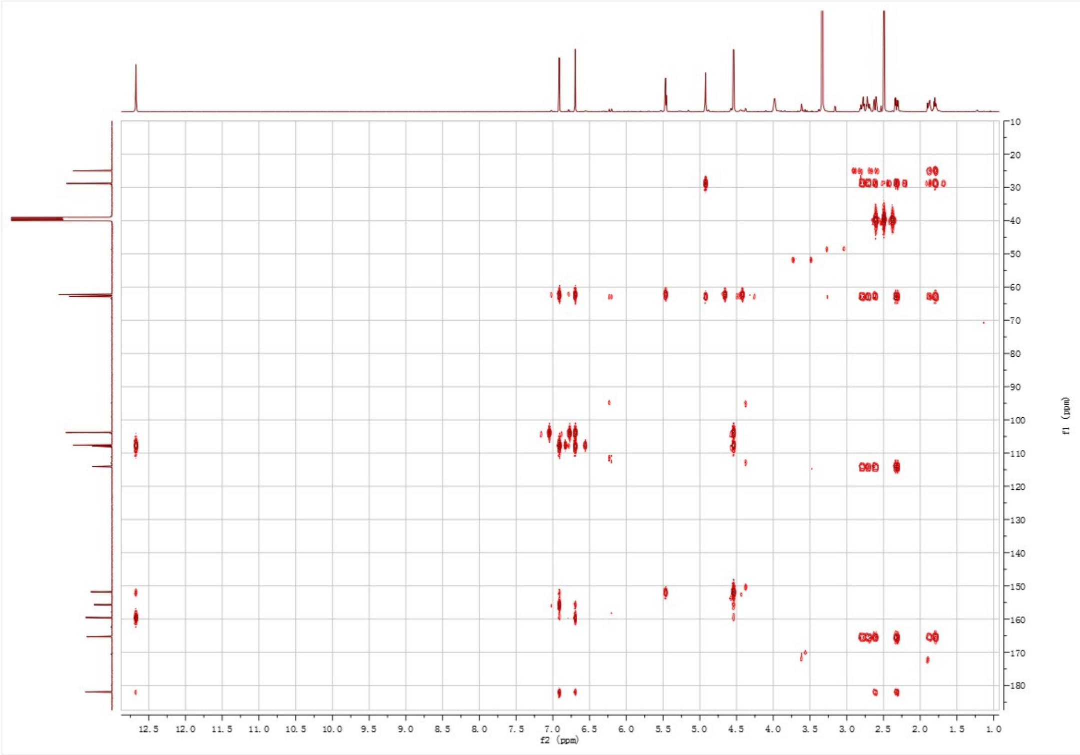

Figure S11. The (+)-HR-ESIMS spectrum of arthone B (**2**)

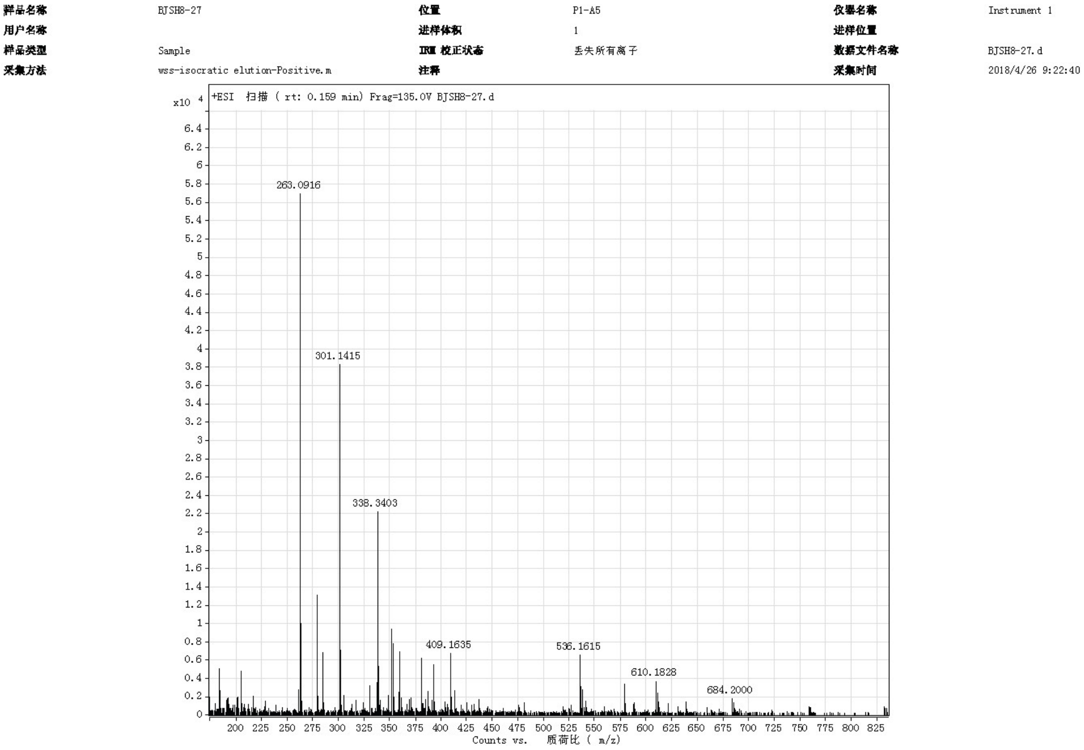

Figure S12. The ^{1}H-NMR spectrum of arthone C (**3**) in DMSO-d_6

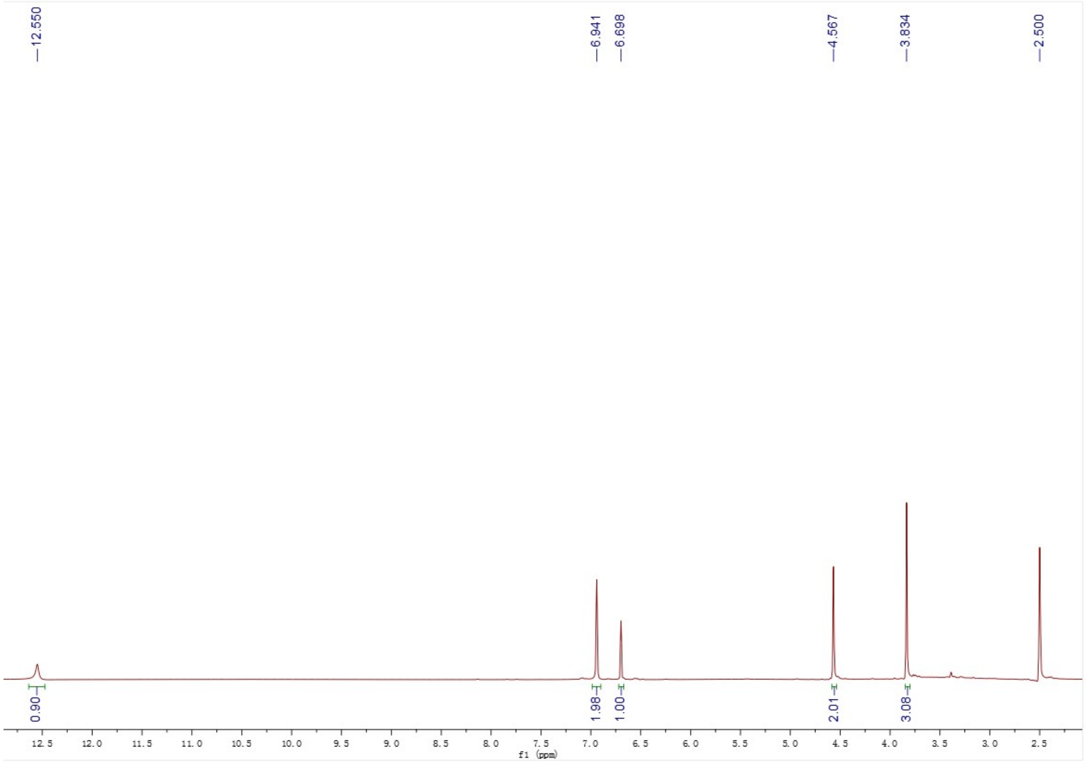

Figure S13. The ^{13}C-NMR spectrum of arthone C (**3**) in DMSO-d_6

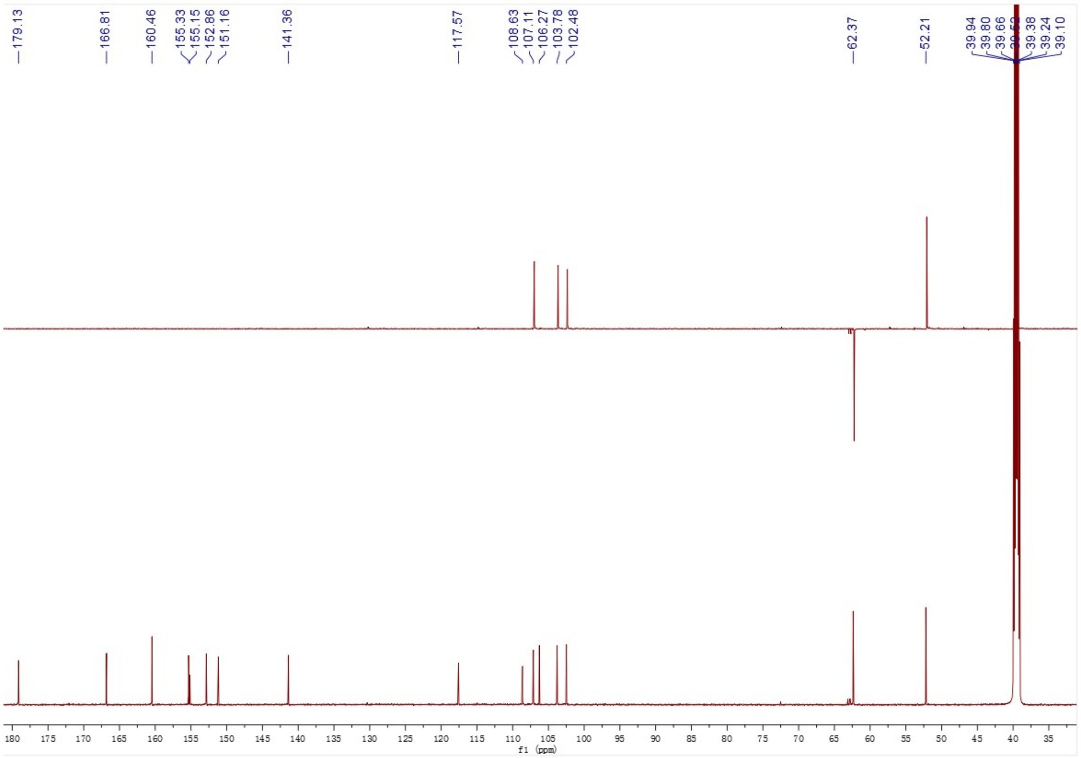

Figure S14. The HSQC spectrum of arthone C (**3**) in DMSO-d_6

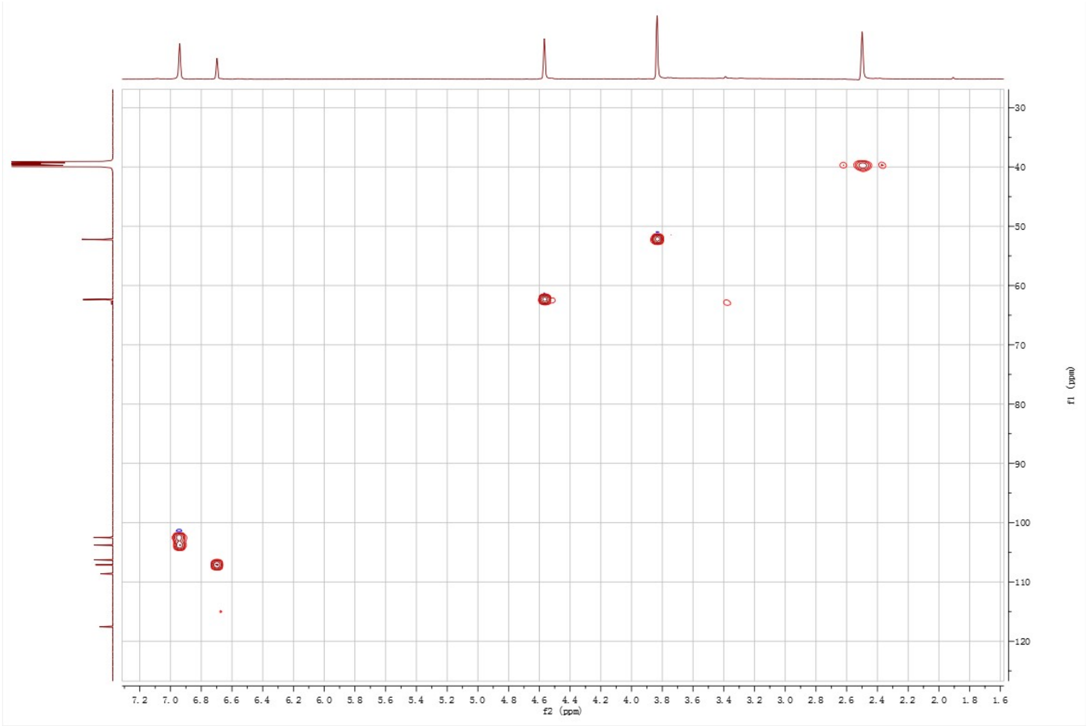

Figure S15. The HMBC spectrum of arthone C (**3**) in DMSO-d_6

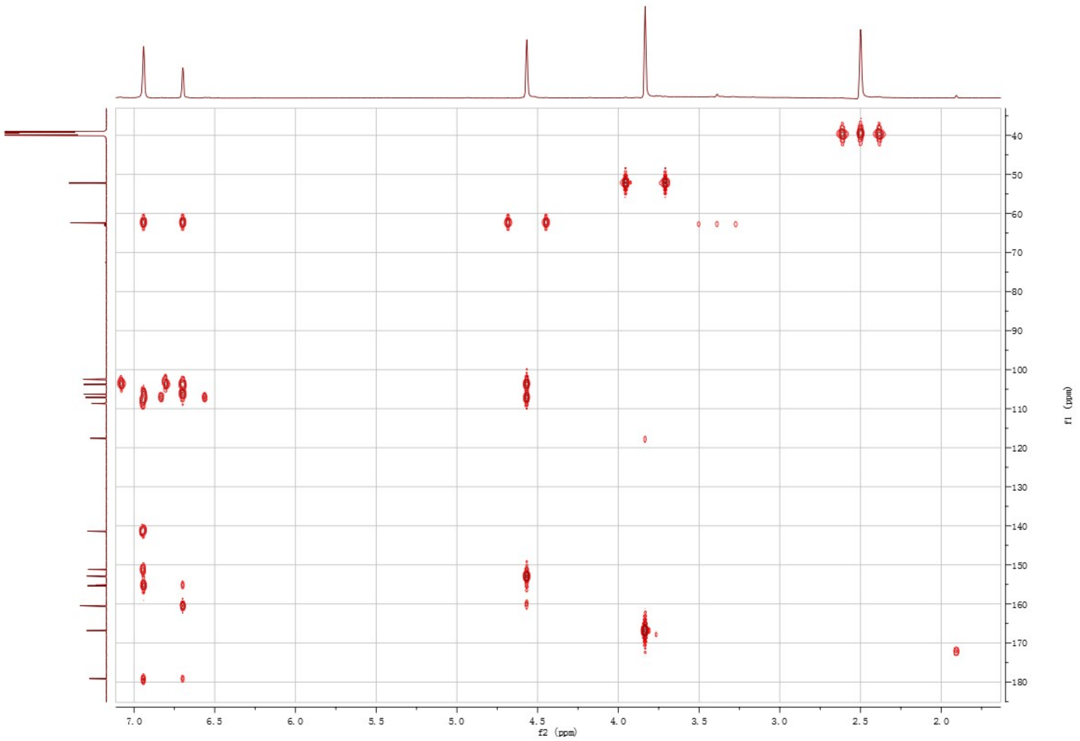

Figure S16. The (+)-HR-ESIMS spectrum of arthone C (**3**)

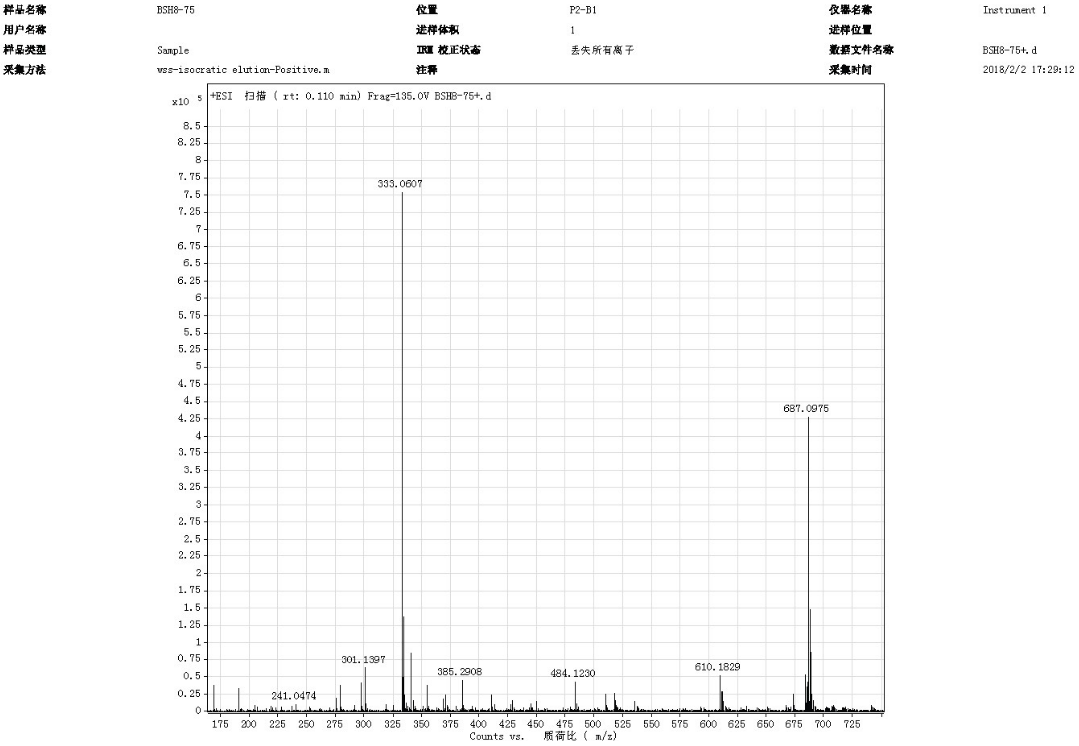

Figure S17. The ^{1}H-NMR spectrum of arthone D (**4**) in DMSO-d_6

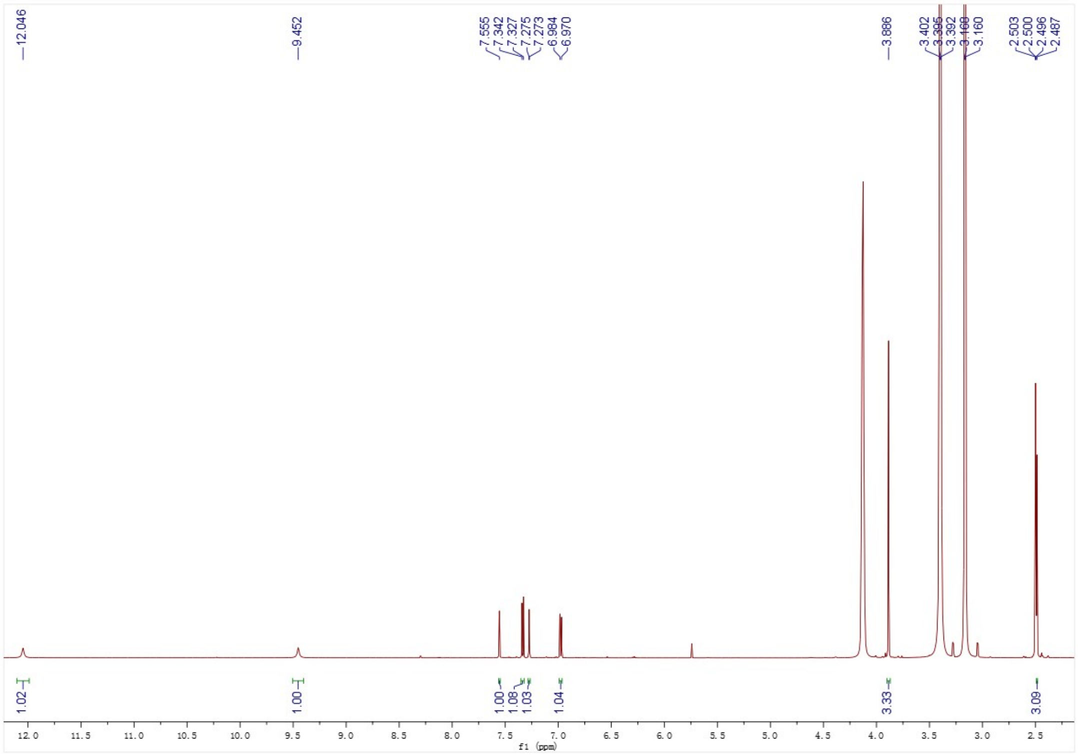

Figure S18. The ^{13}C-NMR spectrum of arthone D (**4**) in DMSO-d_6

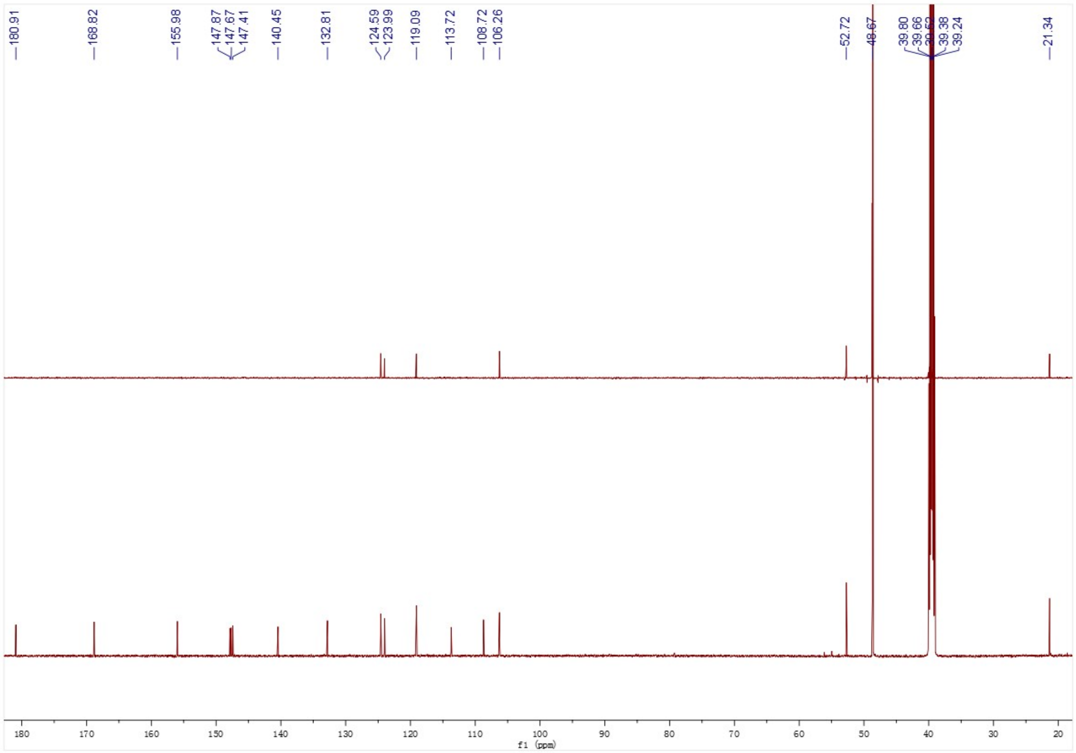

Figure S19. The HSQC spectrum of arthone D (**4**) in DMSO-d_6

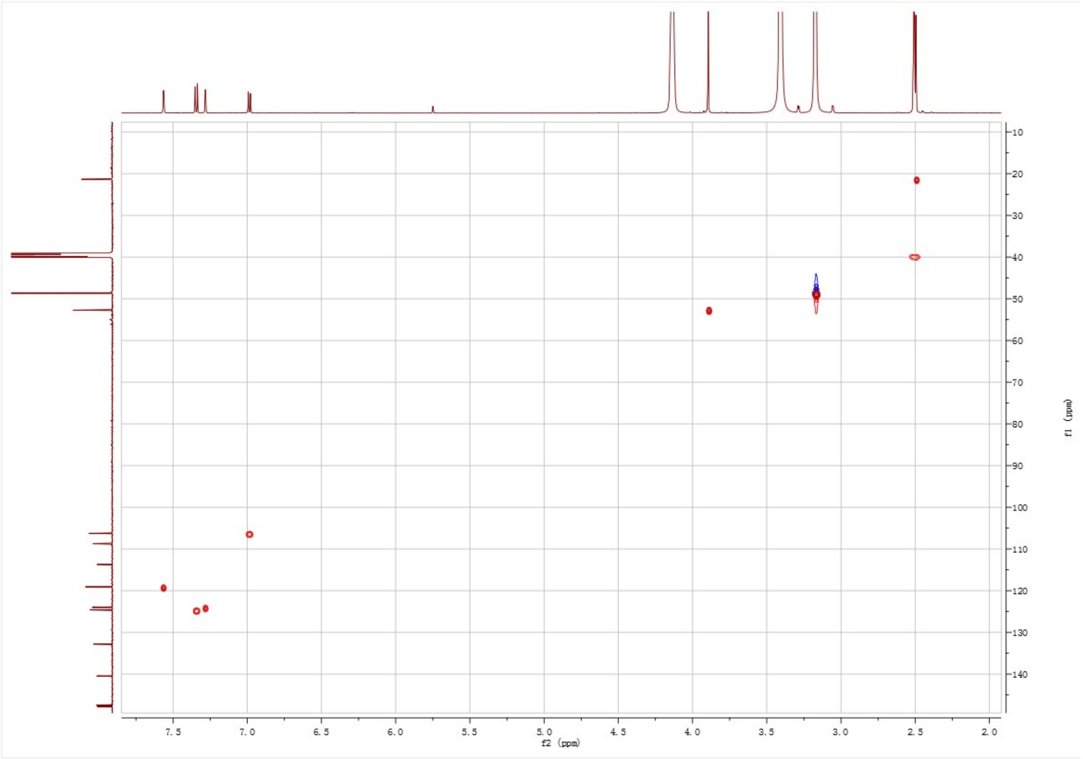

Figure S20. The HMBC spectrum of arthone D (**4**) in DMSO-d_6

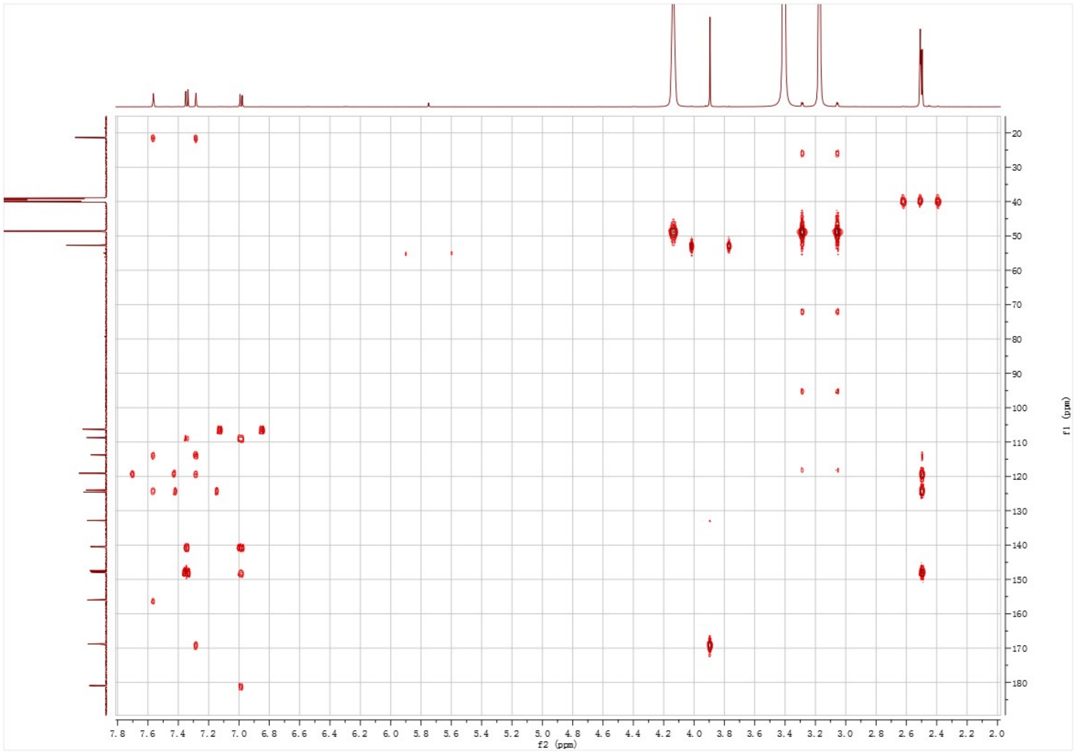

Figure S21. The (+)-HR-ESIMS spectrum of arthone D (**4**)

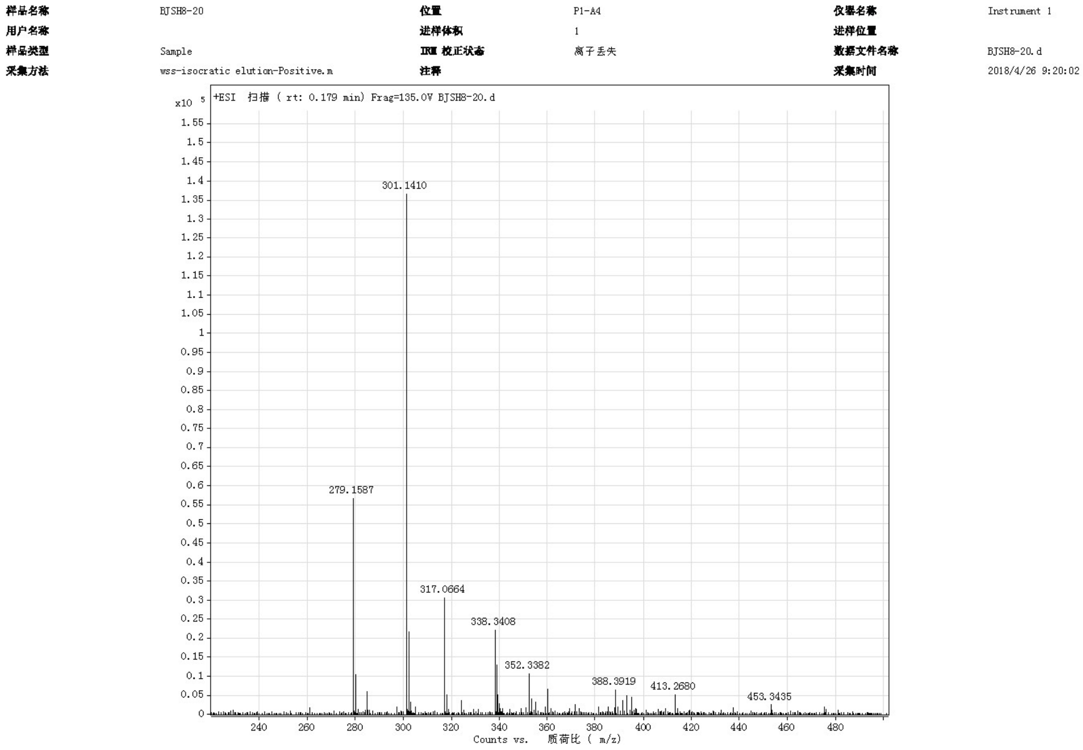

Figure S22. The ^{1}H-NMR spectrum of arthone E (**5**) in DMSO-d_6

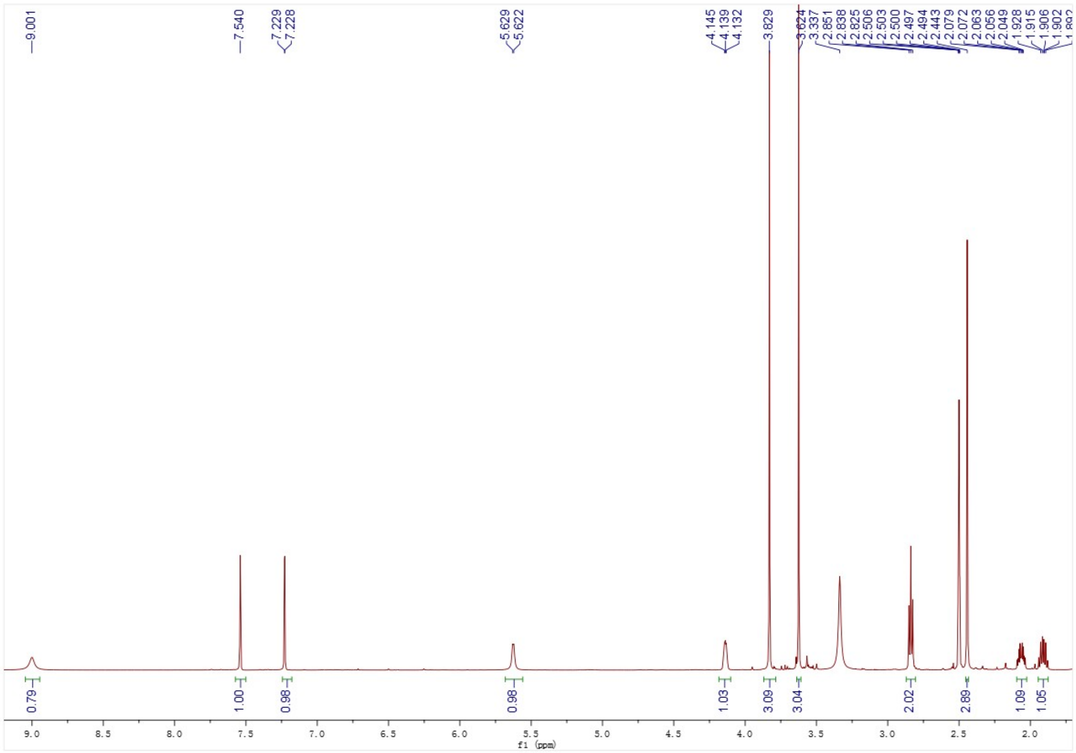

Figure S23. The ^{13}C-NMR spectrum of arthone E (**5**) in DMSO-d_6

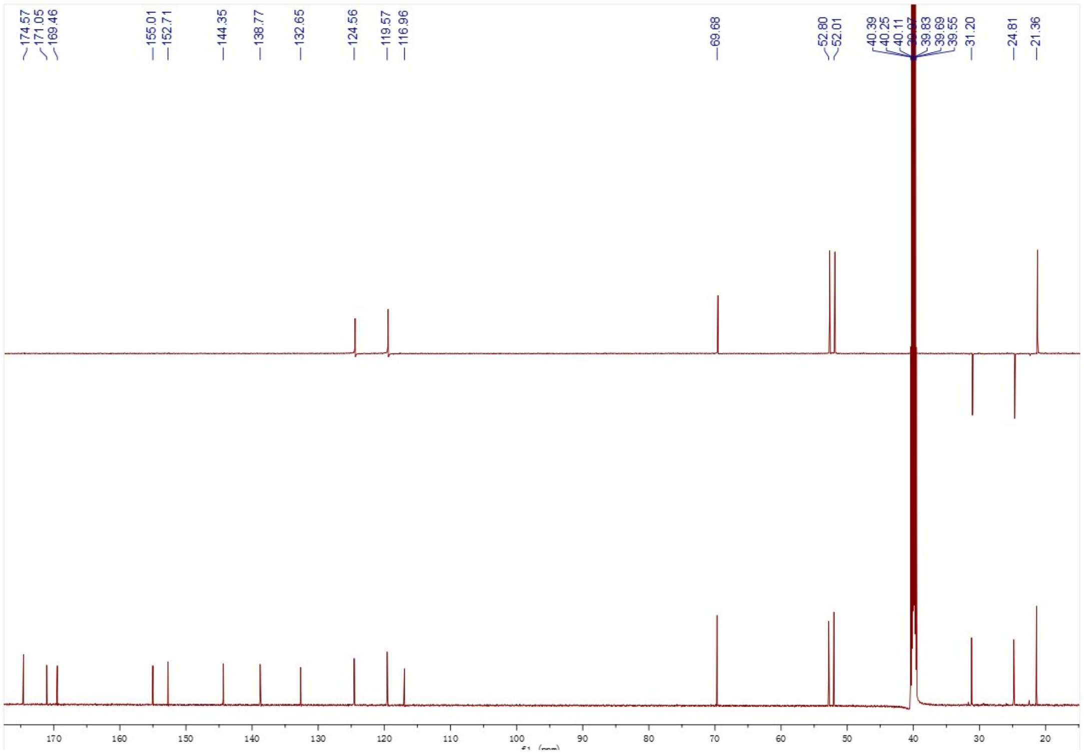

Figure S24. The HSQC spectrum of arthone E (**5**) in DMSO-d_6

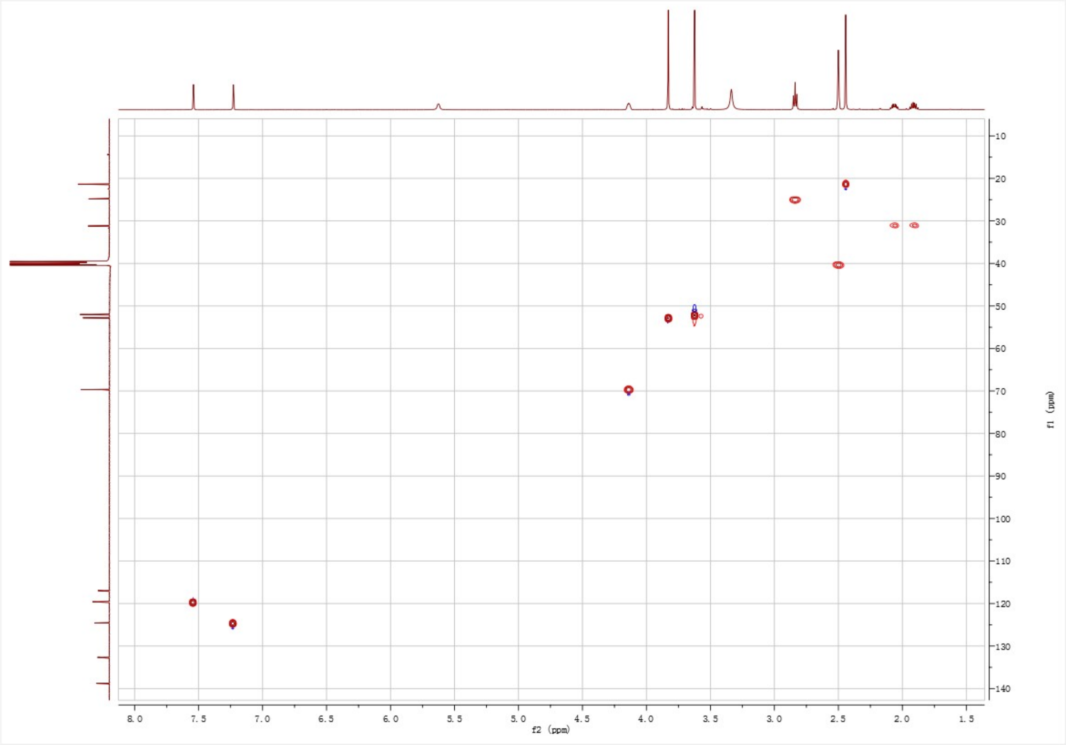

Figure S25. The ^{1}H-^{1}H COSY spectrum of arthone E (**5**) in DMSO-d_6

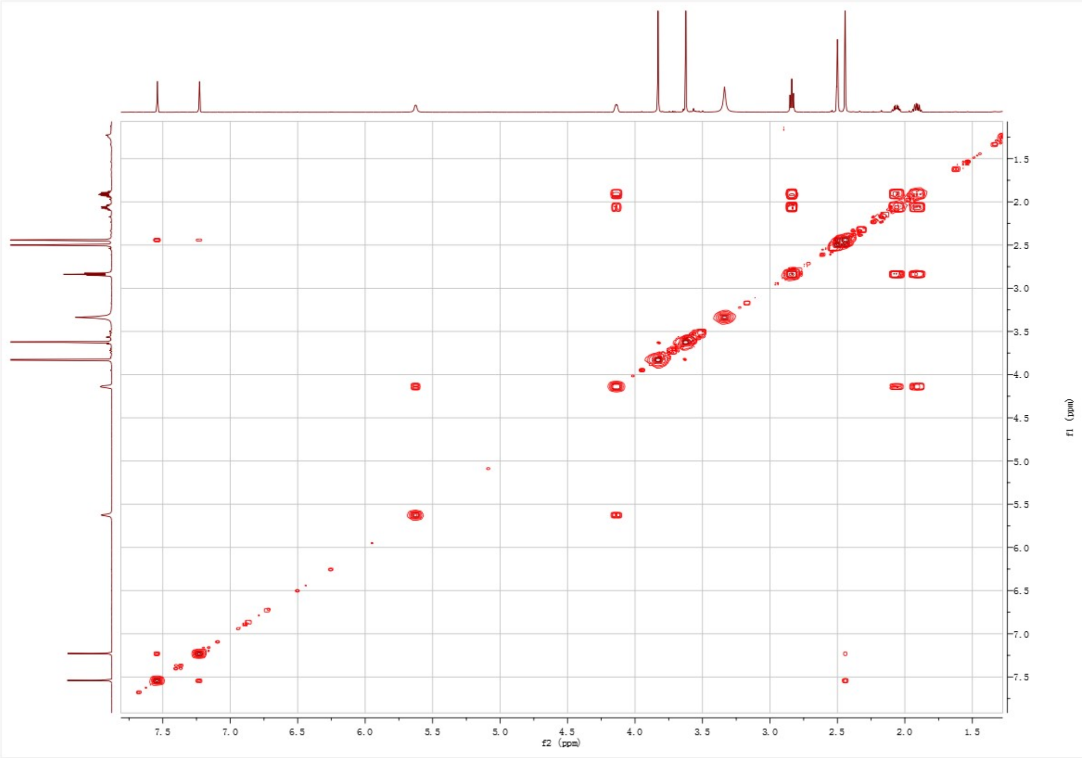

Figure S26. The HMBC spectrum of arthone E (**5**) in DMSO-d_6

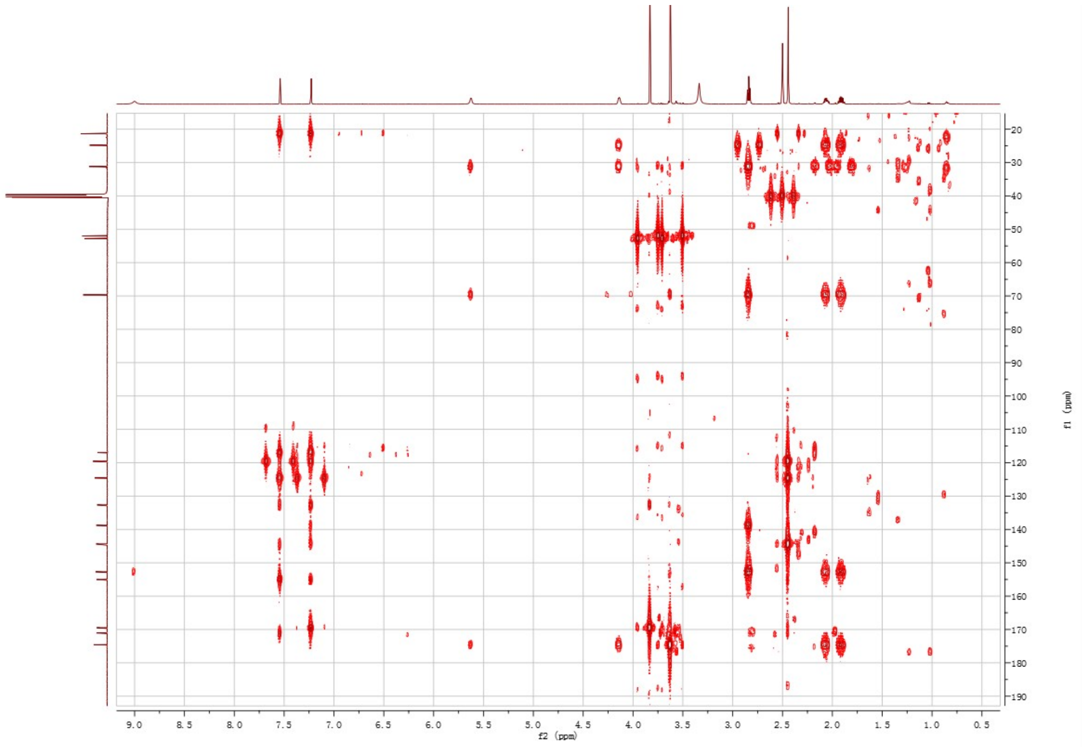

Figure S27. The (+)-HR-ESIMS spectrum of arthone E (**5**)

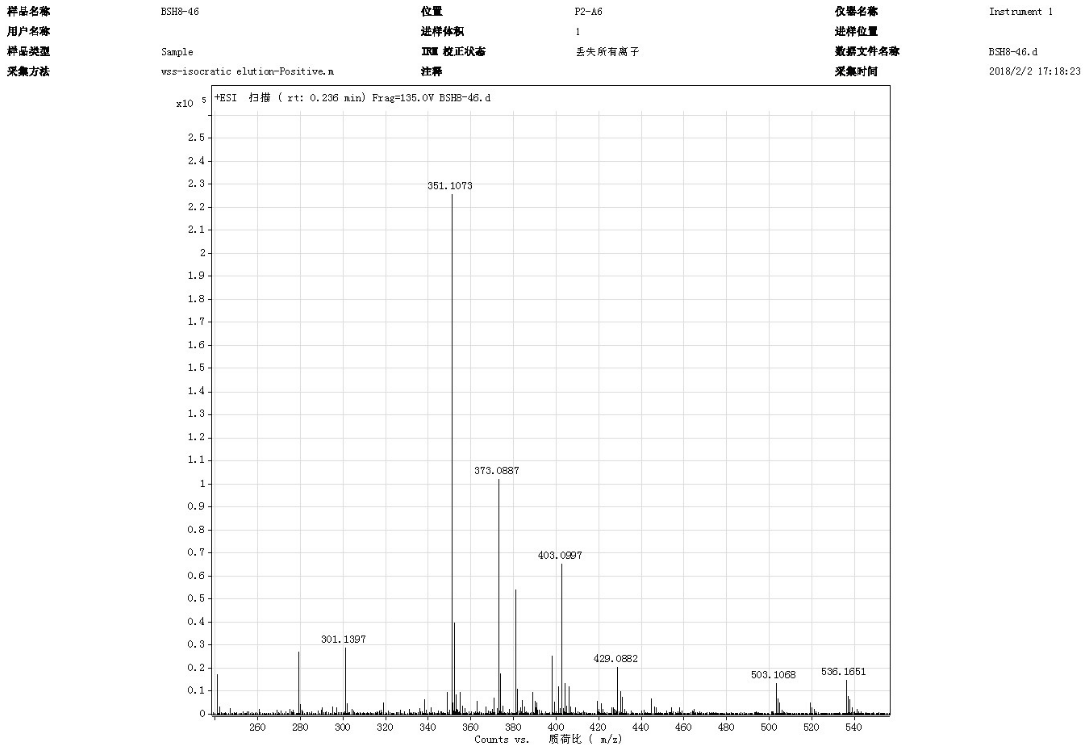

Figure S28. The ^{1}H-NMR spectrum of (*R*)-MTPA ester of arthone B (**2**) in $CDCl_3$

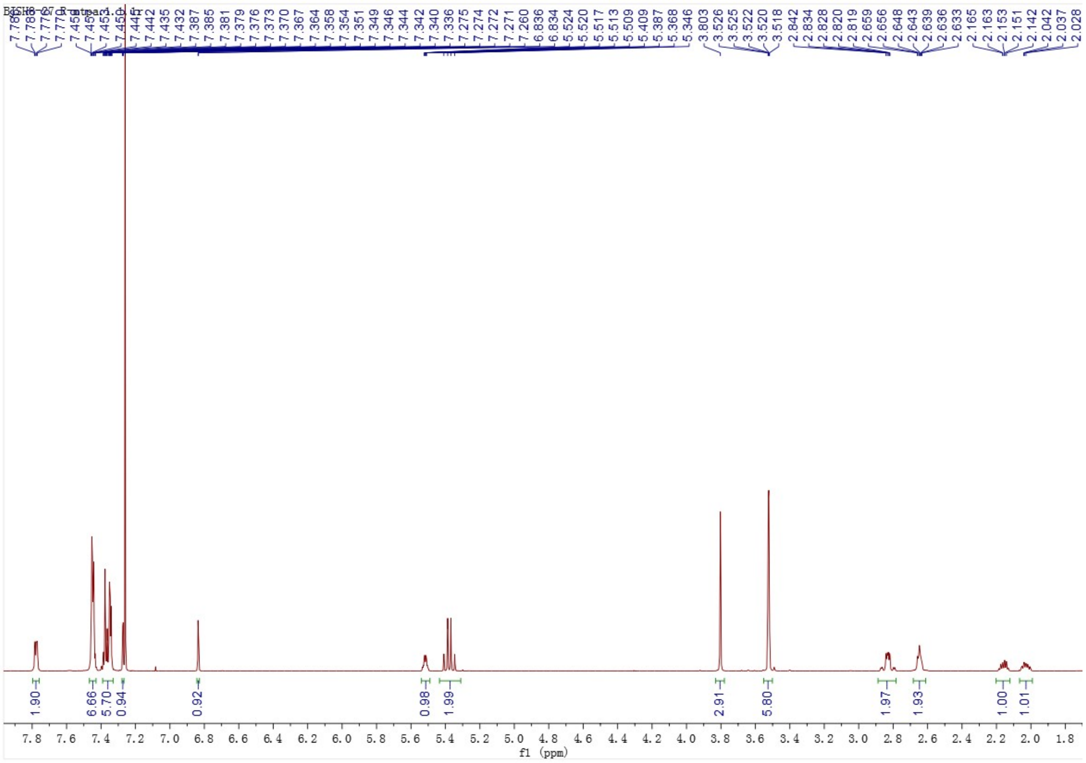

Figure S29. The ^{1}H-^{1}H COSY spectrum of (*R*)-MTPA ester of arthone B (**2**) in $CDCl_3$

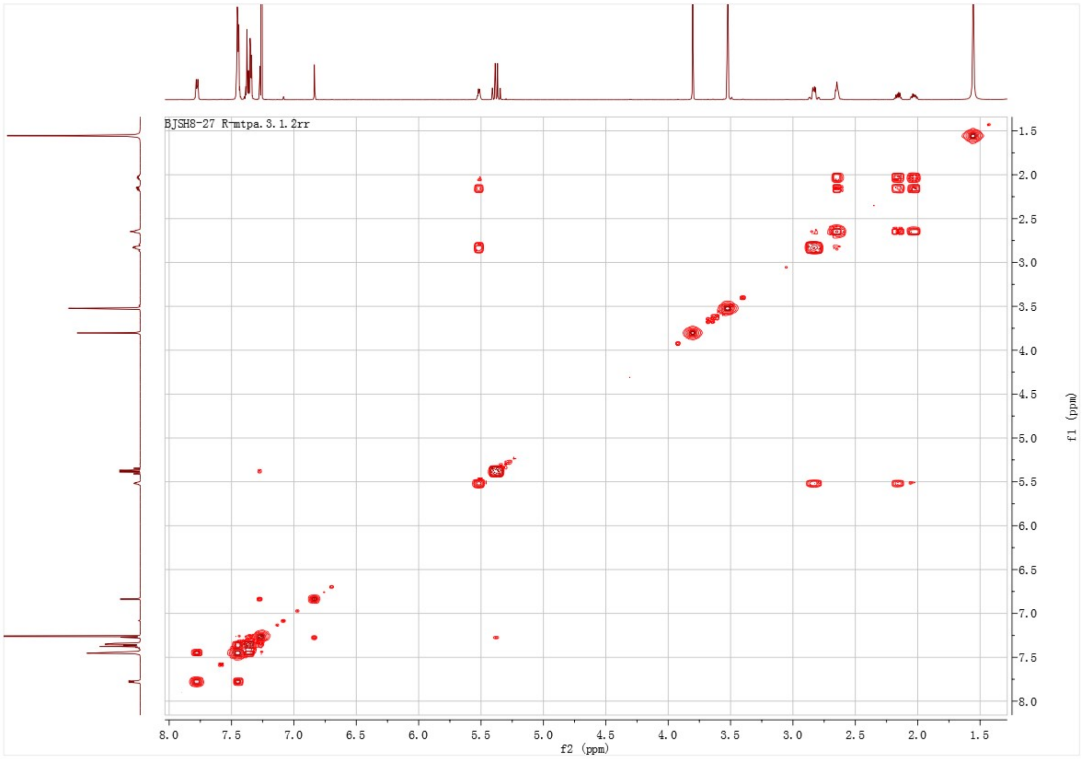

Figure S30. The ^{1}H-NMR spectrum of (*S*)-MTPA ester of arthone B (**2**) in $CDCl_3$

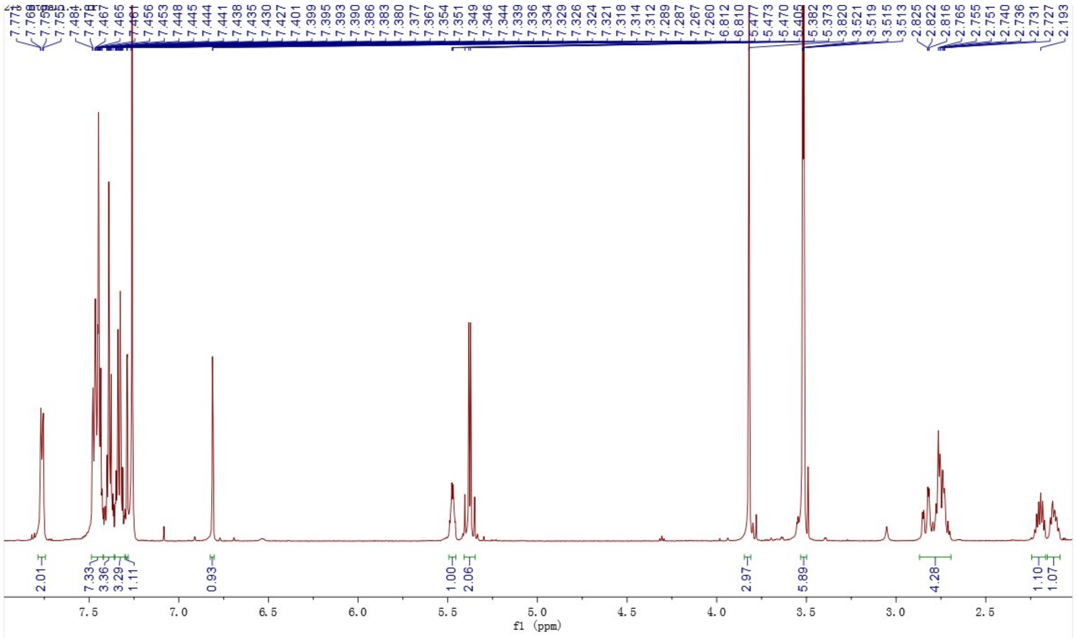

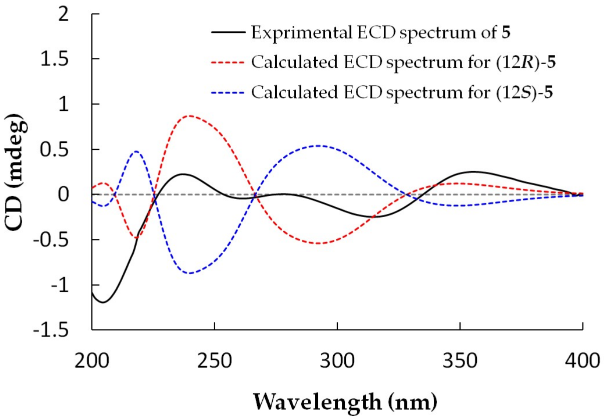

Figure S31. Experimental and theoretical ECD spectra for **5**
